# Modeling the Influence of Coastal Site Characteristics on PFAS in Situ Remediation

**DOI:** 10.1111/gwat.13456

**Published:** 2024-12-11

**Authors:** Grant R. Carey, Anthony Danko, Anh Le‐Tuan Pham, Keir Soderberg, Beth Hoagland, Brent Sleep

**Affiliations:** ^1^ Naval Facilities Engineering and Expeditionary Warfare Center San Diego CA; ^2^ University of Waterloo Waterloo ON Canada; ^3^ S.S. Papadopulos & Associates Rockville MD; ^4^ University of Toronto Toronto ON Canada

## Abstract

The potential performance of a hypothetical colloidal‐activated carbon (CAC) in situ remedy for perfluorooctanoic acid (PFOA) and perfluorooctane sulfonic acid (PFOS) in groundwater in coastal zones was evaluated using estimated hydrogeologic and geochemical parameters for a coastal site in the United States. With these parameters, a reactive transport model (ISR‐MT3DMS) was used to assess the effects of tidal fluctuations and near‐shore geochemistry on CAC performance. The average near‐shore ionic strength of 84 mM at the site was conservatively estimated to result in an increase in the adsorption of PFOA to CAC by about 50% relative to non‐coastal sites with ionic strength <10 mM. The modeling also confirmed the hypothesis that tidally induced groundwater flow reversals near the shore would result in the accumulation of PFOA at the downgradient edge of the CAC zone. Slow desorption of PFOA from this downgradient CAC boundary may sustain downgradient plume concentrations above a strict cleanup criterion (e.g., USEPA MCL of 0.004 μg/L), for decades; however, there was still a large PFOA mass flux reduction (>99.9%) achieved after several decades at the shore. CAC longevity was substantially greater for PFOS with a similar source concentration; however, the higher PFOS distribution coefficient (*K*_*d*_) in soil downgradient from the CAC zone resulted in substantially longer flushing times. It is recommended that short‐term remedial action objectives for CAC remedies at coastal sites be based on mass flux reduction targets over a period of several decades, given the demonstrated challenges in trying to achieve very low cleanup criteria downgradient of a CAC zone in the short term.

## Graphical abstract

This study evaluates the performance of colloidal‐activated carbon barriers for in‐situ remediation of PFAS plumes at coastal sites.

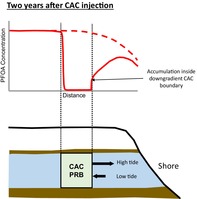

## Introduction

The historical use of aqueous film‐forming foam (AFFF) in fire training activities has resulted in plumes of per‐ and poly‐fluoroalkyl substances (PFAS) in groundwater (Adamson et al. 2020; Leeson et al. 2021; Carey et al. 2022). A promising technology for in situ PFAS remediation involves the injection of colloidal‐activated carbon (CAC) to form a permeable reactive barrier (PRB) that can sequester PFAS (McGregor 2018, 2020a, 2020b, 2023; Carey et al. 2019, 2022, 2023; McGregor and Benevenuto 2021 and McGregor and Zhao 2021). With ongoing PFAS mass flux into a CAC adsorption zone, the activated carbon will eventually become less effective at adsorbing PFAS (i.e., “spent”), resulting in the breakthrough of PFAS downgradient of the CAC zone. Carey et al. (2019, 2022, 2023) indicated that the CAC longevity, defined as the time from CAC PRB installation to target PFAS breakthrough at applicable cleanup criteria, may be on the order of decades; and that this longevity is directly proportional to adsorption strength and inversely proportional to PFAS mass flux into the CAC zone.

Coastal site hydrogeology and the associated geochemistry warrant special attention with respect to the potential for both adverse and beneficial effects on the performance of a CAC remedy in groundwater. For example, groundwater elevations and velocity in the near‐shore area exhibit significant spatial and temporal fluctuations in response to tidal oscillations (Ferris 1952). During low tide, groundwater will flow toward the shore. During high tide, groundwater flow directions will reverse in the near‐shore area such that flow is inward from the shore area.

**Figure 1:**
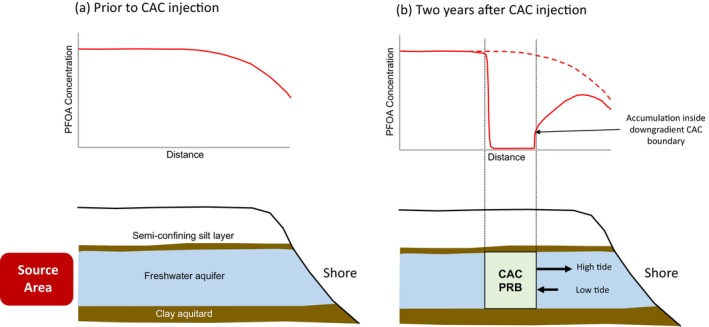
Conceptual model for colloidal activated performance at a coastal site. (a) Prior to injection; and (b) 2 years after injection.

Figure 1a and 1b present a conceptual model for the implementation of a CAC PRB remedy in the near‐shore area at a coastal site with a PFAS plume. Figure 1a illustrates that prior to CAC injection, PFAS is discharging to surface water based on the average regional hydraulic gradient. PFAS concentrations at the shore are diluted due to the diurnal inward flow of clean seawater. The degree of dilution will depend in part on the magnitude of the average regional hydraulic gradient and the tidal amplitude. After the construction of a CAC PRB in the near‐shore area (Figure 1b), PFAS aqueous concentrations will decline by orders of magnitude in the CAC adsorption zone. PFAS concentrations in the area situated between the CAC zone and the shore will also decline over time. In the near‐shore area, however, groundwater flow reversals will cause PFAS to be transported diurnally toward the “downgradient” boundary of the CAC zone. In this study, “downgradient” is defined based on the direction of the average regional hydraulic gradient.

This diurnal reversal of flow direction may result in an increase in PFAS concentrations at the downgradient boundary of the CAC zone as shown in Figure 1b. As groundwater flow reverses again and starts to flow outward to the shore, PFAS will desorb from this downgradient CAC boundary. This cycle of flow reversals is hypothesized to result in sustained PFAS concentrations downgradient of the CAC zone, at a level similar to the PFAS concentration that is attained at the downgradient CAC boundary, for potentially a long period of time. This is one phenomenon at coastal sites which may adversely influence the performance of a CAC remedy.

Groundwater geochemistry in the near‐shore environment at a coastal site is characterized by high ionic strength, as well as high concentrations of divalent ions including calcium and magnesium. Adamson et al. (2022) note that the adsorption of PFAS was enhanced by a factor of 10 at a coastal site, relative to what would be expected for a freshwater aquifer. Elevated ionic strength and concentrations of calcium and magnesium will enhance PFAS adsorption to natural organic matter (NOM) as a result of electrostatic interactions such as the neutralization of soil particle charges and the suppression of intermolecular repulsion between PFAS anions; as well as increased hydrophobic interactions between PFAS and NOM (You et al. 2010; Chen et al. 2012; Cai et al. 2022; Yin et al. 2022). You et al. (2010), Chen et al. (2012), and Oliver et al. (2020) determined that the quantity of PFAS adsorption to NOM was directly proportional to the fraction of organic carbon (*f*_*oc*_) in soil for both freshwater and seawater environments, indicating that PFAS adsorption to mineral surfaces was negligible relative to adsorption to NOM.

Hakimabadi et al. (2023) determined that elevated ionic strength and calcium concentrations will enhance the adsorption of long‐chain PFAS to CAC. Mole et al. (2023) determined that increasing ionic strength resulted in a decline in short‐chain PFAS adsorption to a different type of CAC. It is important to consider how enhanced PFAS adsorption to NOM and CAC may affect remedy performance at coastal sites.

The purpose of this study was to assess the potential effects of coastal hydrogeology and geochemistry on the performance of a hypothetical CAC PRB for PFAS in situ remediation. Hydrogeological and geochemical conditions at a coastal site in the United States provided relevant parameters for developing simplified numerical models to assess these effects. A one‐dimensional groundwater flow model was constructed to generate possible hydraulic head and groundwater velocity fluctuations in the near‐shore environment at the site. A reactive transport model was utilized to investigate the effects of coastal site characteristics on CAC PRB performance. Potential effects on post‐injection PFAS concentrations in the CAC zone, CAC longevity, and the rate of PFAS flushing downgradient of the CAC zone were considered. A sensitivity analysis was conducted to evaluate how the distance between the CAC zone and the shore, the amount of PFAS adsorbed to NOM prior to CAC injection, the PFAS‐CAC Freundlich isotherm, and the effect of tidal fluctuations influence CAC performance. The findings of this study are relevant to future site characterization, feasibility study, remedial design, or remedy optimization at coastal sites with high concentrations of PFAS in groundwater.

## Coastal Site Characterization

**Figure 2:**
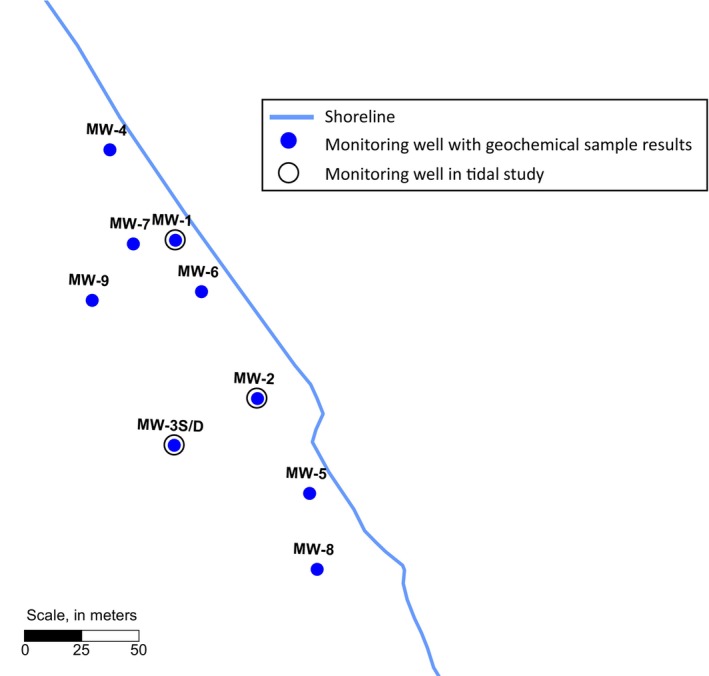
Coastal site monitoring well locations.

Hydrogeological and geochemical conditions used in this study were based on the monitoring of a coastal site in the United States. The purpose of using conditions at this site is to provide relevant parameter values for the assessment of a hypothetical CAC PRB at a coastal site, rather than to develop a comprehensive finely calibrated model for the site. The hydrostratigraphic unit of interest for this study is a shallow, semi‐confined aquifer adjacent to the shoreline. A thin silt layer generally overlies this hydrostratigraphic unit at a depth of approximately 1.5 m below‐ground surface (m bgs), and a thick clay aquitard forms the base of this unit. The average saturated thickness of this shallow hydrostratigraphic unit is about 3 m. The aquifer is comprised of artificial fill which is made up of fine sand with silt close to the shore. Ten monitoring well locations at the site are shown in Figure 2. The results of this coastal site characterization informed the parameterization of the groundwater flow and reactive transport models used to assess the performance of a hypothetical CAC remedy.

### Hydrogeology

Four inland monitoring wells were fitted with transducers for a 3‐week tidal study (MW‐1, MW‐2, MW‐3S, and MW‐3D) that was conducted by others between October 29 and November 19, 2021. As part of this current study, we used the transducer‐based monitoring results from the prior tidal study to estimate the aquifer hydraulic conductivity.

**Figure 3:**
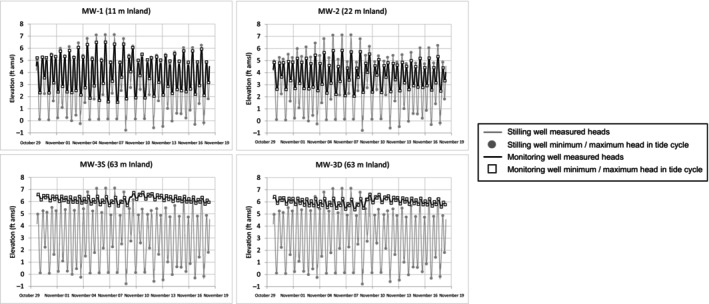
Graphs of tidal monitoring data that were collected as part of a prior study. The minimum and maximum elevations associated with each tidal cycle were determined as part of this current study.

These tidal study monitoring wells are situated at distances inland (*d*_inland_) of 11, 22, and 63 m, respectively. A stilling well in the sea was also monitored as part of this tidal study. Transducer readings were collected at 10‐min intervals at these five wells throughout the 3‐week period. As part of this current study, the minimum and maximum groundwater and seawater elevations corresponding to each diurnal tidal cycle were identified, based on the tidal period of 12.5 h (see Figure 3). The average tidal amplitude (i.e., half of the range between minimum and maximum seawater elevations in the stilling well) is 0.73 m. Tidal efficiency represents the ratio of the range in groundwater elevations in a monitoring well to the range in seawater elevations at the stilling well. Average tidal efficiencies in order of increasing *d*_inland_ were calculated to be 60% (MW‐1), 42% (MW‐2), 7.2% (MW‐9S), and 8.6% (MW‐9D). All four monitoring wells were screened in the shallow artificial fill unit.

Assuming that the average tidal cycle follows a sinusoidal pattern, the hydraulic head in a confined aquifer may be calculated at any point in space and time based on the analytical equation presented in Jacob (1950):

(1) $$\begin{array}{ll} & h\left(d_{\text{inland}},t\right)\\ & \;=h_{o}e^{\left(-d_{\text{inland}}\sqrt{\mathrm{pS}/t_{p}T}\right)}\sin\left(\frac{2\mathrm{pt}}{t_{p}}-d_{\text{inland}}\sqrt{\mathrm{pS}/t_{p}T}\right) \end{array}$$

where *h* is the hydraulic head at *d*_inland_ (m) and time *t* (d), *h*_*o*_ is the tidal amplitude (m), *S* is storativity (dimensionless), *T* is transmissivity (m^2^/d), and *t*_*p*_ is the tidal period (d). Storativity is calculated using *S* = *S*_*s*_ *b*, where *S*_*s*_ is specific storage (1/m) and *b* is saturated thickness (m); and *T* = *Kb* where *K* is hydraulic conductivity (m/d). Literature values of compressibility in Freeze and Cherry (1979) and Domenico and Mifflin (1965) may be used to estimate specific storage for various soil types (Table S1). Assuming that a unit comprised of artificial fill has aquifer compressibility characteristics similar to a loose sand, *S*_*s*_ was estimated for this study to be 6 × 10^−4^ m^−1^.

**Figure 4:**
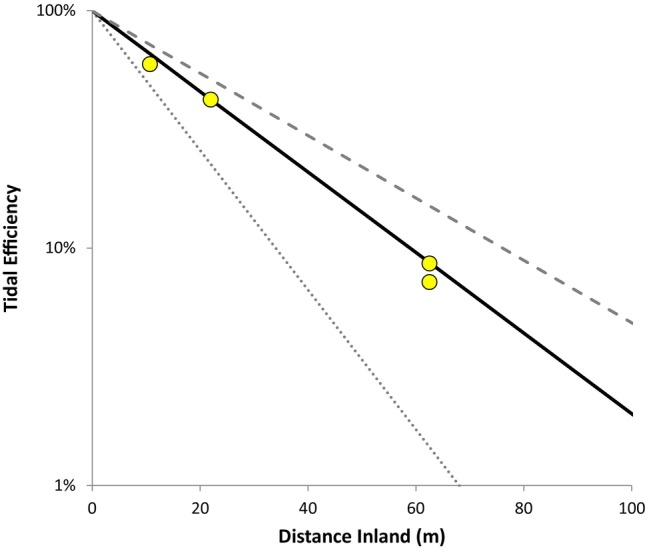
Observed and modeled tidal efficiency versus distance inland. The solid black line represents the calibrated case with hydraulic conductivity (K) of 3 × 10^−5^ m/s; the dotted line represents the modeled case with K of 1 × 10^−5^ m/s; and the dashed line represents K of 5 × 10^−5^ m/s. The symbols represent observed data for MW‐1, MW‐2, MW‐3S, and MW‐3D.

While amplitude and phase lag analyses (e.g., Carr and Van Der Kamp 1969) can be used to estimate hydraulic conductivity in tidal zones, we opted for a simple approach for this study, consistent with study objectives. Hydraulic conductivity in the monitored portion of the aquifer was estimated by matching the modeled and observed tidal efficiency versus distance curves. The analytical solution in Equation 1 was used with the various tidal parameters listed above to model the fluctuating hydraulic heads at each monitoring well location (Figure S1). The modeled tidal efficiency at each monitoring well was then calculated using the modeled range in heads at each monitoring well location, relative to the average range in seawater head at the stilling well. Figure 4 shows the tidal efficiency versus distance curve for the calibrated hydraulic conductivity of 3 × 10^−5^ m/s (2.6 m/d), which matches reasonably well to the observed tidal efficiency versus distance trend. Additional data series are plotted for K = 1 × 10^−5^ and 5 × 10^−5^ m/s to illustrate that the tidal efficiency versus distance curve is sensitive to *K*. In addition to matching the observed tidal efficiency versus distance trend, the calibrated *K* of 2.6 m/d is consistent with the description of lithology in the artificial fill (i.e., mainly fine‐grained sand with silt).

Serfes (1991) indicated that the average regional hydraulic gradient in a tidally influenced environment may be estimated based on the difference in average heads between two monitoring wells. Based on the average heads at the four closest monitoring wells to the shore that were monitored during the tidal study (MW‐27, MW‐28, MW‐9S, and MW‐9D), the average regional hydraulic gradient was estimated to be 0.015 m/m. Given that there was a large rain event 1 week prior to the tidal study, it is assumed for the purpose of this study that the annual average regional hydraulic gradient is approximately one‐half of the gradient measured during the tidal study period (i.e., 0.0075 m/m).

### Geochemistry

Geochemical parameters measured in groundwater at this coastal site include alkalinity, calcium (Ca^2+^), chloride (Cl^−^), magnesium (Mg^2+^), potassium (K^+^), sodium (Na^+^), sulfate (SO_4_^2−^), dissolved organic carbon (DOC), total organic carbon (TOC), and total suspended solids (TSS). Results for these geochemical parameters at the 10 monitoring wells are summarized in Table S2. Near‐shore wells were defined for this study to be those wells within 25 m of the shore, to facilitate the calculation of average geochemistry in the area where the hypothetical CAC zone was to be emplaced for this study. Average near‐shore concentrations for each of the geochemical parameters are listed in Table 1.

**Table 1:** Average Geochemical Analytical Results for Five Wells in the Near‐Shore Area

Parameter	Near‐Shore Average Result	Units
Ionic strength	84	mM
Alkalinity	400	mg/L
Sodium + chloride	3300	mg/L
Potassium	69	mg/L
Calcium	190	mg/L
Magnesium	190	mg/L
Sulfate	260	mg/L
DOC	9.1	mg/L
TOC	9.1	mg/L
TSS	7.8	mg/L

Note: The near‐shore area is defined as monitoring wells situated within 25 m of the shore. This includes monitoring wells MW‐1, MW‐2, MW‐4, MW‐5, and MW‐6 (see Figure 2).

The sum of chloride + sodium represents a proxy for salinity, where seawater typically has chloride + sodium of approximately 35,000 mg/L. The average chloride + sodium for the near‐shore wells at this site is 3300 mg/L, which represents about 10% of the value in seawater; this confirms that groundwater in the artificial fill hydrostratigraphic unit has a density comparable to that of freshwater. Ionic strength (*I*) was calculated at each monitoring well based on the equation:

(2) $$\begin{array}{ll} I & =\left[\left({\mathrm{Cl}}^{-}\right)+\left({\mathrm{Na}}^{+}\right)+\left(K^{+}\right)+4\left({\mathrm{Ca}}^{2+}\right)\right.\\ & \;+\left.4\left({\mathrm{Mg}}^{2+}\right)+4\left({\mathrm{SO}}_{4}^{2-}\right)\right]/2 \end{array}$$

Concentration versus *d*_inland_ scatter plots for ionic strength, chloride + sodium, calcium, magnesium, sulfate, and DOC are shown in Figure S2. These scatter plots indicate that *I* and chloride + sodium are high in the near‐shore area, and these properties decrease with increasing distance inland. There is also a decline in calcium and magnesium concentrations with increasing distance inland, which indicates that PFAS adsorption to NOM in the near‐shore environment will be stronger than at distances further inland. There is no apparent trend in the DOC scatter plot.

The average ionic strength was 84 millimoles per liter (mM) in the near‐shore area, which is near the high range of ionic strength (100 mM) determined by Hakimabadi et al. (2023) to result in increased PFAS adsorption to CAC. The typical ionic strength in seawater is 700 mM, indicating that groundwater in the near‐shore aquifer is diluted with about 10% seawater. The lowest ionic strength of 7 mM was measured at monitoring well MW‐9 which is situated 55 m inland. Chen et al. (2012) determined the effect on PFOS *K*_*d*_ from varying dilutions of seawater and freshwater at neutral pH in a single soil sample. Assuming that freshwater at the site has *I* of 7 mM (i.e., the minimum measured at MW‐9), we can calculate the ionic strength for the seawater–freshwater mixtures used by Chen et al. (2012) with 0%, 10%, 25%, and 50% seawater; these mixtures correspond to ionic strengths of 7, 76, 180, and 354 mM, respectively.

In this current study, the “relative *K*_*d*_” is defined as the ratio of PFOS *K*_*d*_ at a specific seawater/freshwater mixture, to the PFOS *K*_*d*_ in 100% freshwater. The relative *K*_*d*_ for 100% freshwater in this case is 1.0. Using the *K*_*d*_ data presented in the study by Chen et al. (2012), we calculate relative *K*_*d*_ values as follows: 3.6 in a mixture with 10% seawater, 7.1 with 25% seawater, and 9.8 with 50% seawater. These data are illustrated in Figure S3, and they represent the factors of *K*_*d*_ increases as the seawater fraction increased in the solution mixture. At the coastal site in this current study, groundwater in the near‐shore area is mixed with approximately 10% seawater, which indicates that PFOS adsorption to NOM may be enhanced by a little more than three times relative to adsorption occurring in freshwater further inland.

DOC and TOC had similar average near‐shore concentrations (Table 1), indicating that organic carbon in groundwater was dissolved. The DOC concentrations (8.1 to 11.15 mg/L) are higher than 5 mg/L and are thus expected to have some competitive influence on PFAS adsorption to CAC (Carey et al. 2022; Hakimabadi et al. 2023; Mole et al. 2023).

## Modeling Approach for Evaluating CAC Performance

### General Approach

For the purpose of generating transient groundwater flow velocities for assessment of PFAS transport and sequestration by CAC a one‐dimensional groundwater flow model was constructed using MODFLOW (Harbaugh and McDonald 1996). The one‐dimensional flow model was assumed to be adequate for the purposes of this study of a hypothetical coastal zone CAC PRB. Consequently, the effects of aquifer heterogeneity, leakage through the overlying silt layer, and vertical flow were neglected. A model domain length of 300 m was specified to ensure that there was negligible tidal influence at the upgradient domain boundary. This domain length was confirmed using the analytical solution in Equation 1. The downgradient boundary of the model domain represented transient head oscillations occurring at the groundwater–surface water interface at the shoreline.

**Figure 5:**
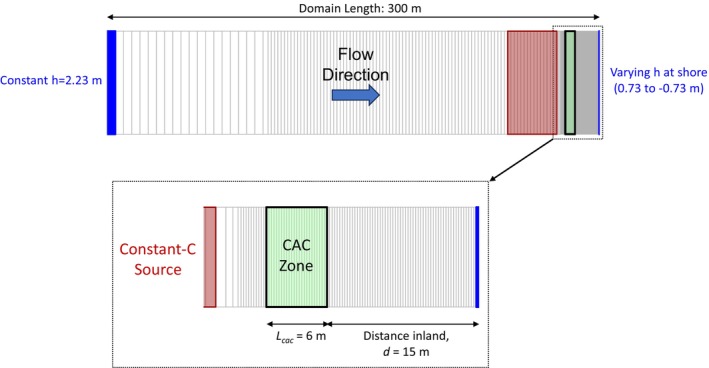
Model domain extent and grid discretization.

The model domain, boundary conditions, and spatial discretization are shown in Figure 5. For this modeling study, the average seawater elevation was specified to be 0 masl. The upgradient boundary was specified to have a constant‐head of 2.23 masl, which results in an average regional hydraulic gradient of 0.0075 m/m. The average groundwater flow direction in the model is from left to right in Figure 5. The downgradient constant‐head boundary was specified to have sinusoidal head oscillations with a tidal amplitude of 0.73 m and a tidal period of 12.5 m. Grid spacing was 0.25 m in the downgradient region of the model domain, and ranged up to 5 m in the upgradient region.

The simulated hydraulic heads and groundwater velocities were utilized with the In‐Situ Remediation Model (ISR‐MT3DMS), a proprietary reactive transport model capable of simulating multispecies transport in one‐, two‐, or three‐dimensions (Porewater Solutions 2022). ISR‐MT3DMS was constructed to simulate advection using the MT3DMS Total Variation Diminishing (TVD) scheme based on the explicit finite difference method with upstream weighting. Carey et al. (2019, 2022, 2023) document the numerical formulation that has been incorporated into ISR‐MT3DMS for simulating CAC performance and longevity for in situ PFAS remediation.

In the current study, 1D transport simulations were conducted. Consequently, diffusive losses to the overlying and underlying formations are neglected and dilution due to transverse dispersion that may have a significant effect on narrow plumes is also neglected. These simplifications maximize the predicted flux of perfluorooctanoic acid (PFOA) into the CAC zone and into the downgradient area compared to a more complex 3‐D simulation.

PFOA was selected as the target constituent for evaluating CAC performance. Carey et al. (2022) indicate that the two most common PFAS of concern (POCs) are PFOA and PFOS. PFOS typically adsorbs more strongly to CAC than PFOA, and thus PFOA will be the POC that will exhibit the earliest breakthrough at many sites (Carey et al. 2022).

Carey et al. (2023) documented the maximum PFOA groundwater concentration in the source area at a South Dakota site to be 321 μg/L. Based on the statistical analysis of maximum PFOA concentrations at 96 AFFF‐impacted sites (Carey et al. 2022), Carey et al. (2023) determined that PFOA concentrations at that South Dakota site were higher than at many other AFFF‐impacted sites. For the hypothetical CAC remedy scenario in this current study, a constant PFOA source concentration of 300 μg/L was specified to represent worst‐case conditions relative to most AFFF‐impacted sites. As the source concentration may be expected to decline over the lifetime of the CAC PRB, this is an additional worst‐case condition.

A more detailed discussion of the model construction and verification is described below.

### Groundwater Flow Model Construction and Calibration

Eight stress periods were used to represent the oscillating head boundary conditions for each 12.5‐h tidal period at the groundwater‐surface water interface in the flow model (Figure S4). The hydraulic heads at the transient head boundary were estimated using the analytical solution in Equation 1 with *d*_inland_ = 0.125 m, which is the inland distance of the last, rightmost node in the model grid. The heads modeled with this analytical solution are shown in Figure S4. The MODFLOW boundary condition heads corresponding to the start of each stress period are also shown in Figure S4. Each stress period had a uniform duration of 0.0651 days, and each stress period was divided into 10 time steps of uniform duration. The MODFLOW Time‐Variant Specified‐Head Package was used to represent transient head conditions at this downgradient boundary. The PCG2 solver was used, and the residual head and flow criteria were specified to be 1 × 10^−5^ m and 1 × 10^−4^ m^3^/d, respectively. The mass balance error at the end of the 1‐year groundwater flow simulation with 5584 stress periods was 0.02%.

To demonstrate that the numerical model was representative of the analytical model solution results, the MODFLOW‐simulated heads were compared to the analytical solution results for *d*_inland_ of 15, 30, and 60 m. In the model domain, *d*_inland_ is measured from the right domain boundary. Figure S5 shows that the MODFLOW simulation results are close to the analytical model results, indicating that the constructed MODFLOW model is providing a reasonable representation of tidally influenced groundwater head fluctuations estimated with the analytical solution.

**Figure 6:**
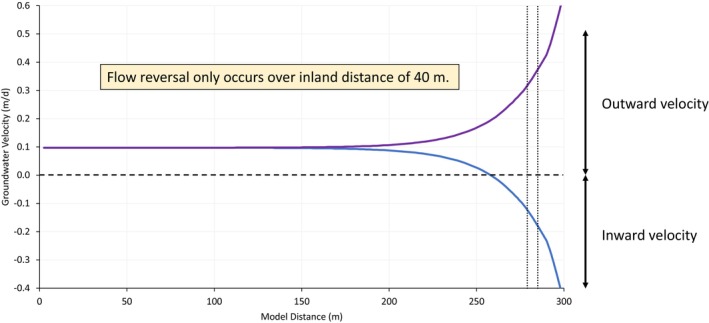
Simulated minimum and maximum average linear groundwater velocity during each tidal cycle. The purple line series represents the maximum velocity, and the blue line series represents the minimum velocity at each distance. The horizontal dashed line represents a velocity of zero, and the vertical dotted lines represent the extent of the simulated CAC zone. The downgradient CAC zone boundary is at a distance inland of 15 m. Groundwater velocity below the horizontal dashed line is inward from the coast; and velocity above the dashed line is outward. The simulated velocities indicate that inward flow only occurs intermittently during the tidal cycle up to a distance inland of 40 m.

The simulated minimum and maximum groundwater velocities for the assumed conditions are shown in Figure 6, based on an effective porosity of 0.20. This effective porosity represents the mid‐point of the range of 0.1 to 0.3 estimated for fine sand in USEPA (1998). The base case location for the hypothetical CAC zone in the reactive transport model is also shown in Figure 6. The groundwater velocity at the downgradient boundary of the CAC zone, which corresponds to *d*_inland_ = 15 m, ranges from a maximum inward velocity of −0.18 m/d to a maximum outward velocity of 0.36 m/d. Figure 6 illustrates that the predicted minimum and maximum groundwater velocities increase exponentially with decreasing distance to the shoreline, which is consistent with what is expected for this type of environment. Figure 6 illustrates that tidally influenced groundwater fluctuations occur up to *d*_inland_ of 125 m; however, the groundwater flow reversals (i.e., where groundwater velocity becomes negative) only occur up to *d*_inland_ of 40 m. This indicates that with the conditions determined for this specific coastal site and the associated model assumptions and simplifications, a CAC zone installed at *d*_inland_ of more than 40 m would not be subject to reversals in the direction of groundwater flow. Beyond this distance the groundwater flow direction at this site is predicted to be consistently outwards toward the shore.

### Reactive Transport Model Construction

At coastal sites, it is important to consider the enhanced adsorption of PFAS to NOM that occurs due to elevated ionic strength and divalent cation (i.e., calcium and magnesium) concentrations. Adamson et al. (2022) measured a perfluorohexane sulfonic acid (PFHxS) distribution coefficient (*K*_*d*_) of 1.03 mL/g at a coastal site, which is about 10 times higher than the *K*_*d*_ of 0.13 mL/g that would be calculated using a PFHxS organic carbon partitioning coefficient (*K*_*oc*_) of 130 mL/g (Carey et al. 2019) and the fraction of organic carbon (*f*_*oc*_) reported by Adamson et al. (2022) for the coastal site (0.1%). Similarly, Adamson et al. (2022) measured a *K*_*d*_ of 10.1 mL/g for PFOS. The 10 times ratio of *K*_*d*_ values for PFOS and PFHxS is generally consistent with their relative adsorption affinities to NOM at non‐coastal sites (e.g., based on a *K*_*oc*_ of 920 mL/g for PFOS as determined by Carey et al. 2019). The *K*_*oc*_ values summarized by Carey et al. (2019) are specific to measurements from one site and are generally consistent with ranges of *K*_*oc*_ observed at other sites.

PFHxS and PFOA have similar *K*_*oc*_ properties (e.g., see Carey et al. 2019, table S2; Chen et al. 2016; Navarro et al. 2022), so PFOA may be expected to have a similar *K*_*d*_ to PFHxS at coastal sites. The median *f*_*oc*_ at the coastal site considered in this current study is 0.3%. Using a *K*_*oc*_ value of 120 mL/g for PFOA, this corresponds to a theoretical *K*_*d*_ of 0.36 mL/g. For the base case model scenario of PFOA transport in the near‐shore environment, a *K*_*d*_ of 1.2 mL/g was specified to account for the increase in *K*_*d*_ observed with PFOS in the Chen et al. (2012) study for a 10% seawater solution (see Figure S3). This enhanced *K*_*d*_ represents an *equivalent* minimum *f*_*oc*_ of 1.0%, relative to the actual *f*_*oc*_ of 0.3%. The difference between the equivalent *f*_*oc*_ and the actual *f*_*oc*_ represents the effects of enhanced electrostatic and hydrophobic interactions in the near‐shore environment.

The extent and location of the hypothetical PFOA source area, where a constant‐concentration boundary was specified at 300 μg/L, is shown in Figure 5. While the source of PFOA likely originated at the ground surface above the silt confining layer, for the purpose of simplicity, it was assumed that the source area of interest was located entirely in the aquifer simulated by the flow model. The ISR‐MT3DMS transport model was first used to simulate the pre‐injection PFOA concentrations that represent a dynamic equilibrium in the tidally influenced near‐shore area. Dynamic equilibrium refers to the condition where tidally influenced concentration oscillations become steady over time between tidal cycles. This dynamic equilibrium condition was reached at the right model boundary after several months into a 1‐year simulation period. The right model boundary represented specified heads only; it was not used as a constant‐concentration boundary. The ISR‐MT3DMS model assumes that an inward flux of water into the domain has a concentration of zero at a specified‐head boundary, and that the outgoing water will have the same concentration as that simulated at the boundary cell.

The PFOA concentrations corresponding to the last time step in this pre‐remediation model scenario were used as the initial condition for model simulations that represented the injection and performance of a CAC PRB. For the pre‐remediation simulation, the average PFOA concentration at the shore was predicted to be approximately 150 μg/L, which represents a dilution factor at the groundwater‐surface water interface of about 50% relative to the source area concentration. This dilution factor is sensitive to the regional average hydraulic gradient; for example, if the hydraulic gradient is reduced by a factor of 10 in the model, then the modeled PFOA concentration at the groundwater‐surface water interface is diluted by 90% relative to the source area concentration.

All transport model simulations were conducted for a 1‐year simulation period, which corresponds to the same simulation period for the groundwater flow model. When evaluating CAC performance after injection, it was necessary to simulate PFOA transport for periods of up to 40 years. A system of Fortran pre‐ and post‐processors was used to construct the initial concentration input matrix for each 1‐year simulation, based on the simulated PFOA concentrations at the end of the previous year in another model run. Batch programs were used to automate the sequential simulation of PFOA transport after CAC injection for simulation periods of either 30 or 40 years, depending on the model input parameters used for each scenario.

ISR‐MT3DMS simulates PFAS adsorption as follows: 1. Prior to CAC injection, a linear isotherm is represented throughout the model domain; after injection, the Freundlich isotherm is used in the CAC zone to represent PFAS adsorption (Carey et al. 2022). At the time of CAC injection in the model, ISR‐MT3DMS uses a mass balance approach (Carey et al. 2019) to estimate the re‐equilibration of PFAS mass between the aqueous phase, adsorption to CAC, and adsorption to NOM in the PRB. This re‐equilibration step facilitates the representation of post‐injection PFAS concentrations in the PRB, and is simulated in ISR‐MT3DMS as an instantaneous step.

In this study, PFAS adsorption to CAC is represented based on equilibrium conditions. As shown in Figure 6, the simulated groundwater velocity in the PRB ranges from approximately −0.2 m/day (inward) to 0.4 m/day (outward). The extent to which PFAS adsorption to CAC in these velocity conditions may exhibit non‐equilibrium behavior is uncertain; given that these minimum and maximum velocities occur only over a short duration of the tidal cycle, it may be reasonable to represent the average PRB performance based on equilibrium conditions. More research is needed to verify this, however.

The adsorbed concentration, *S* (mg/kg) is determined using *S* = *f*_*cac*_ *K*_*f*_ *C* ^*a*^, where *f*_*cac*_ is the fraction of CAC in soil (g/g), *K*_*f*_ is the Freundlich adsorption coefficient (mg/kg[mg/L]^−*a*^), *a* is the Freundlich isotherm exponent (dimensionless) which represents the degree of non‐linearity in the isotherm, and *C* is the aqueous concentration (mg/L). A simple mass balance equation may be used to estimate the initial aqueous PFAS concentrations immediately after CAC injection, using a Freundlich isotherm to represent PFAS partitioning to CAC (Carey et al. 2019):

(3) $$C_{\text{post}}={\left[\frac{C_{o}\left(q+K_{\mathrm{oc}}f_{\mathrm{oc}}r_{b}\right)}{K_{f}f_{\mathrm{cac}}r_{b}}\right]}^{1/a}$$

where *C*_*o*_ and *C*_post_ are the pre‐ and post‐injection aqueous PFAS concentrations (mg/L), *θ* is effective porosity (m^3^/m^3^), and *ρ*_*b*_ is the soil dry bulk density (g/mL). This mass balance assumes that PFAS mass will be predominantly re‐partitioned as adsorbed to CAC over NOM, and thus it may not be valid to use Equation 3 to estimate post‐injection concentrations for short‐chain PFAS or where *f*_*oc*_ is high. Refer to Carey et al. (2019, 2022, 2023) for a more detailed description of the ISR‐MT3DMS numerical algorithm used to simulate CAC performance.

At present, the PFOA adsorption isotherm with CAC has only been derived for one non‐coastal field site where AFFF impacts had occurred; Carey et al. (2022) report a PFOA *K*_*f*_ = 580 mg/kg (mg/L)^−*a*^, and *a* = 0.25 based on a groundwater sample collected from PFAS impacted groundwater at this site. This PFOA‐CAC isotherm represents competitive adsorption effects related to the presence of high concentrations of DOC, PFOS, and other PFAS in the groundwater sample (see Carey et al. 2022). For the coastal site in this current study, we need to adjust this isotherm to represent the influence of increased ionic strength in the near‐shore environment. Hakimabadi et al. (2023) estimated PFOA Freundlich isotherms with CAC for a series of single PFAS‐species batch tests where *I* was controlled to be 0, 1, 10, and 100 mM. Using the four PFOA isotherms specified in Hakimabadi et al. (2023) and an aqueous PFOA concentration of 0.3 μg/L which is consistent with the Hakimabadi et al. (2023) batch test results, the calculated adsorbed PFOA concentrations are shown in Figure S6. Based on this fixed aqueous concentration, a power model regression was derived to estimate the PFOA adsorbed concentration as a function of ionic strength (*S* = 1132 *I* ^0.1363^). The ionic strength of the groundwater sample used to derive the field‐scale PFOA‐CAC Freundlich isotherm was estimated to be 4 mM for a site in South Dakota based on geochemical parameters characterized in Carey et al. (2019), and the average ionic strength in the near‐shore environment at the coastal site is 84 mM. For modeling CAC performance at the coastal site, the PFOA *K*_*f*_ from the field‐scale isotherm was multiplied by the ratio of the adsorbed PFOA concentration (*S*) calculated based on ionic strengths of 84 and 4 mM, resulting in an adjusted *K*_*f*_ of 870 mg/kg (mg/L)^−*a*^ for the coastal site (assuming no change in the Freundlich exponent of 0.25). This represents a 50% increase in PFOA adsorption in the CAC zone due to ionic strength effects.

For the base case scenario, it was conservatively assumed that the high concentrations of calcium and magnesium did not enhance PFOA adsorption in the CAC zone. Hakimabadi et al. (2023) and Carey et al. (2023) do show a significant increase in PFOA adsorption to CAC when the calcium concentration is increased in synthetic laboratory solutions; however, it is uncertain how much effect calcium and magnesium will have on PFOA adsorption to CAC at the field‐scale, where geochemical reactions may be quite different than those occurring in a synthetic laboratory solution (Carey et al. 2023). The influence of using a higher PFOA *K*_*f*_ to consider the potential influence of high calcium and magnesium is assessed as part of the sensitivity analysis presented below.

To represent CAC in the model, it was assumed that CAC was injected 10 days into the remediation simulation period. The *f*_*cac*_ was specified to be 0.8%, which was the maximum *f*_*cac*_ reported for 17 field sites summarized in Carey et al. (2022). The length of the CAC zone (parallel to the regional groundwater flow direction) was specified to be 6 m. The total duration of the base case model scenario was 30 years, and is based on sequential 1‐year transport simulations using the approach discussed above. The ISR‐MT3DMS transport time steps were the same as the flow model time step of 0.00651 days. The base case transport model mass balance error was 0.03% at the end of the 30‐year simulation.

Irvine et al. (2021) discuss the artificial upstream dispersion which occurs in solute transport models. Carey et al. (2023) used local‐scale dispersivity in the CAC zone to reduce the potential for artificial upstream dispersion at the downgradient CAC boundary for a site scenario where groundwater velocity was at steady‐state. A similar approach was used in this study, where a local‐scale dispersivity of 0.2 m (i.e., 3% over 6 m PRB width) was specified in the CAC zone and in the region 1 m upgradient and downgradient from the CAC zone; the dispersivity was simulated to be 2 m (i.e., 10%) over the remaining distance between the source zone and the shore, to reflect the likelihood for increased dispersivity in a tidally influenced environment and over a larger scale.

The base case scenario simulates PFOA transport for a 30‐year period following injection of the CAC PRB. The initial condition for the transport model represents the PFOA plume at dynamic equilibrium with the tidal cycle, prior to CAC injection. The model simulates CAC injection at a simulation time of 10 days, followed by 30 years of post‐injection performance. The base case scenario is based on a PFOA *K*_*d*_ of 1.2 mL/g, a CAC Freundlich coefficient of *K*_*f*_ of 870 mg/kg (mg/L)^−*a*^, and a distance of 15 m between the downgradient CAC zone boundary and the shore.

Six alternative scenarios were simulated as part of a sensitivity analysis, with input parameter adjustments from the base case scenario as follows:

- Case 1: Steady‐state average regional groundwater velocity of 41 m/y to facilitate a comparison to the tidally influenced velocity simulated in the base case scenario;
- Case 2a: *K*_*d*_ = 0.36 mL/g to represent conditions at a non‐coastal site;
- Case 2b: *K*_*d*_ = 2.4 mL/g to represent stronger adsorption to NOM that may occur at a coastal site;
- Case 3: CAC zone moved 5 m closer to the shore with *d*_inland_ = 10 m at the CAC downgradient boundary, to evaluate the effect of a larger inward velocity at high tide; and
- Case 4: *K*_*f*_ = 1740 mg/kg (mg/L)^−*a*^ to represent the potential for enhanced PFOA adsorption to CAC that may occur with the high calcium and magnesium concentrations in the near‐shore environment.
- Case 5: Simulation of a PFOS source instead of PFOA (same concentration), with comparison to the simulated base case PFOA trends at the CAC downgradient boundary and at the shore.

A summary of all model input parameters is presented in Table S3.

For this hypothetical study, the longevity of the CAC PRB will be assessed based on the USEPA MCL of 0.004 μg/L. Note, however, that the actual cleanup criteria applicable at coastal sites may be focused on ecological receptors, in which case the criteria will be higher than MCLs. CAC longevity will not differ significantly with changes in the applicable criteria when equilibrium PFAS adsorption to CAC is simulated, because the PFAS breakthrough curve over time is relatively steep under this condition.

## Model Results and Discussion

### Base Case

**Figure 7:**
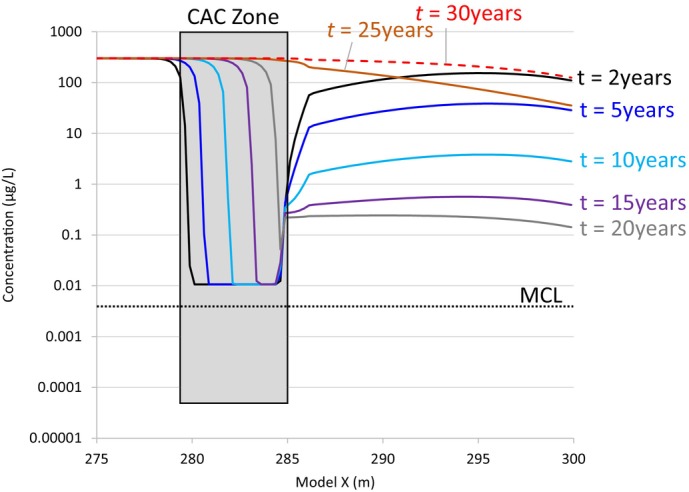
Simulated PFOA concentration versus distance for the base case scenario, at simulated times of 2, 5, 10, 15, 20, 25, and 30 years after CAC injection. The simulated CAC zone is in the gray‐shaded area. The surface water boundary occurs at *x* = 300 m in the model. Groundwater flow direction in the CAC zone is outward toward the shore during low tide, and inwards from the shore at high tide. The increase in PFOA concentrations at the downgradient CAC boundary is due to the transport of PFOA into this boundary during periods of inward flow. Simulated PFOA breakthrough in the CAC zone occurs at a time of about 20 years after CAC injection.

The PFOA concentration versus distance profiles for simulation times of 2, 5, 10, 15, 20, 25, and 30 years after CAC injection are shown in Figure 7 for the base case scenario. The CAC zone upgradient boundary is at model *X* = 279 m, and the downgradient boundary is at *X* = 285 m which corresponds to *d*_inland_ = 15 m. The PFOA concentration upgradient of the CAC zone is steady at 300 μg/L, and the post‐injection PFOA concentration through most of the CAC zone is 0.01 μg/L. This corresponds to a concentration reduction in most of the CAC zone of 99.997%. The concentration at the CAC downgradient boundary is larger, however, due to the inward velocity that is simulated to occur (up to 0.18 m/d) around high tide. PFOA concentrations between the CAC zone and the shore are much higher than the concentration in the CAC zone; when groundwater flow reversal occurs, the inward velocity will result in the accumulation of PFOA on CAC at the downgradient boundary of the CAC zone.

Figure 7 illustrates that downgradient of the CAC zone, the PFOA concentrations decline from a maximum of a little over 100 μg/L at a simulation time of 2 years, down to about 0.2 μg/L at a simulation time of 20 years after CAC injection. This shows that even after 20 years, the PFOA concentration between the CAC zone and the shore continues to be close to two orders of magnitude above the USEPA MCL of 0.004 μg/L. The slow flushing of this zone is due to the enhanced adsorption of PFOA to NOM that occurs where ionic strength and calcium/magnesium levels are high; it takes time for PFOA mass to desorb from NOM and eventually be flushed out at the shore. This PFAS flushing time is inversely proportional to the average linear groundwater velocity through the region between the CAC zone and the shore; sites with higher velocity will experience reduced flushing times.

At a time of 20 years after CAC injection, the PFOA concentration is highest at the downgradient boundary of the CAC zone (model *X* = 285 m), and the concentration gradually declines with decreasing distance toward the shore (model *X* = 300 m). This indicates that PFOA is desorbing from the downgradient CAC boundary and is sustaining PFOA concentrations at slightly lower levels in the region between the CAC zone and the shore. Figure 7 indicates that at the time of 20 years, the PFOA front in the CAC zone has almost penetrated to the downgradient CAC zone boundary, indicating that CAC has become spent after about 20 years in the base case scenario. The PFOA concentration versus distance curves for simulation times of 25 and 30 years after CAC injection indicate that PFOA breakthrough has occurred and conditions are returning to the pre‐remediation condition. Figure S7 shows concentration contours using a color scale for these different time periods, to better illustrate the penetration of the PFOA front through the CAC zone over time.

### Case 1—Comparison to Steady‐State Velocity

**Figure 8:**
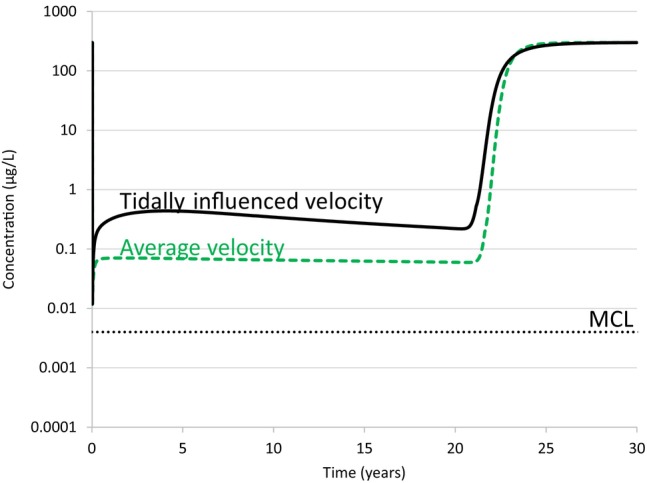
Simulated PFOA concentration versus time at the CAC downgradient boundary for the Base Case with tidal fluctuations, and Case 1 with steady‐state velocity.

To illustrate the effect of tidally influenced velocity on CAC performance, the Case 2 scenario is based on a steady‐state groundwater velocity of 41 m/y which is based on the regional average hydraulic gradient. The PFOA concentrations versus time at the downgradient CAC zone boundary (model *X* = 285 m, *d*_inland_ = 15 m) are compared for the base case and Case 2 in Figure 8. This figure shows that immediately after CAC injection, the PFOA concentration at this downgradient CAC boundary declined to the post‐injection concentration of 0.01 μg/L. PFOA then increased at this CAC downgradient boundary in both cases; in the steady‐state (outward only) velocity scenario (Case 2), PFOA increased at this downgradient boundary to about 0.07 μg/L due to artificial upstream dispersion from the adjacent plume. This artifact is discussed further in Irvine et al. (2021) and Carey et al. (2023). In the base case scenario, the PFOA concentration increased to a maximum concentration of about 0.4 μg/L. While part of the increase in the base case scenario may be due to artificial upstream dispersion, most of this increase at the CAC boundary is due to the inward groundwater velocity that occurs at high tide.

### Case 2—Influence of PFOA K_d_

**Figure 9:**
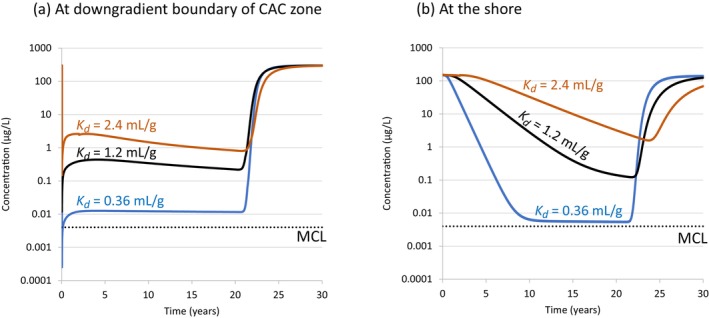
Simulated PFOA concentration versus time at: (a) the CAC downgradient boundary; and (b) the shore. Three simulations are shown: Base Case (*K*_*d*_ = 1.2 mL/g); Case 2a (*K*_*d*_ = 0.36 mL/g); and Case 2b (*K*_*d*_ = 2.4 mL/g).

The simulated PFOA concentration versus time at the downgradient CAC zone boundary are shown in Figure 9a for the base case (*K*_*d*_ = 1.2 mL/g), Case 2a (*K*_*d*_ = 0.36 mL/g) and Case 2b (*K*_*d*_ = 2.4 mL/g). Similarly, the simulated concentrations versus time at the shore are shown in Figure 9b. These results indicate that the PFOA concentration at the downgradient CAC boundary is sensitive to *K*_*d*_. Increasing *K*_*d*_ by about half an order of magnitude (0.36 to 1.2 mL/g) resulted in an increase in the maximum PFOA concentration at this boundary by 1.5 orders of magnitude. The maximum PFOA concentration at the downgradient CAC boundary, in order of increasing *K*_*d*_, was simulated to be 0.01, 0.44, and 2.6 μg/L for *K*_*d*_ values of 0.36, 1.2, and 2.4 mL/g, respectively. The large difference in downgradient CAC concentrations for the low and middle *K*_*d*_ cases is probably due to the substantially faster decline in PFOA that occurs downgradient of the CAC zone for the lower *K*_*d*_ case (see Figure 9b). This faster plume flushing results in a faster reduction in the influx of PFOA into this CAC boundary for the lower *K*_*d*_ case (i.e., when the groundwater flow direction is inward).

The initial decline half‐life in PFOA concentrations at the shore (Figure 9b) was calculated to be 0.5, 1.5, and 3 years for *K*_*d*_ equal to 0.36, 1.2, and 2.4 mL/g, respectively. This demonstrates that the half‐life for declining PFOA concentrations at the shore is directly proportional to *K*_*d*_, as would be expected. While these decline half‐lives have some uncertainty because they are based on the assumption of a linear, fully reversible isotherm for PFOA adsorption to NOM, the general trends are likely representative of what will occur at coastal sites. For example, Chen et al. (2016) determined that PFOA sorption in sediments from an urban reservoir was almost completely reversible; and Navarro et al. (2022) found a higher degree of reversibility for PFOA and PFHxS relative to PFOS, and that there was a small irreversible fraction for PFOA in sediments with high *f*_*oc*_ and long contact times.

Navarro et al. (2022) suggest that some PFAS mass may become physically entrapped within pores of the NOM matrix over time, due to interactions with inner groups of the NOM matrix that lead to irreversible deformations. It is also possible that what may appear to be irreversibly adsorbed in a laboratory experiment over a limited timeframe is actually exhibiting very slow desorption kinetics for a fraction of originally adsorbed PFAS mass. Regardless, the net effect would be similar with respect to the time required to flush PFAS from a region downgradient of a CAC PRB: very slow kinetic desorption or irreversible adsorption will both lead to quicker declines in PFAS concentrations over time, relative to the case where adsorption is fully reversible and desorption occurs at a consistent rate or under equilibrium conditions.

For the low *K*_*d*_ case (i.e., non‐coastal site), the shoreline PFOA concentration declined quickly from about 150 μg/L (pre‐remediation) to a level that was just over the USEPA MCL, and then the decline essentially ceased. This is because PFOA is desorbing from the downgradient CAC boundary (Figure 9a), which resulted in sustaining PFOA concentrations above the MCL between the CAC zone and the shore (Figure 9b) for the low *K*_*d*_ case. For example, the PFOA concentration at the downgradient CAC boundary just prior to breakthrough was 0.011 μg/L for the low *K*_*d*_ case (Figure 9a). The simulated PFOA concentration reached a plateau at the shoreline at a concentration of about 50% of this upgradient concentration, due to attenuation from inter‐tidal mixing at the shore. This demonstrates how PFOA accumulation at the downgradient CAC boundary may sustain a low‐level plume between the CAC PRB and the shore.

The shore concentration versus time curves for the higher *K*_*d*_ scenarios (i.e., coastal site scenarios) did not plateau like the low *K*_*d*_ case. PFOA breakthrough at the downgradient boundary of the CAC zone occurred at around 20 years for all *K*_*d*_ simulations (Figure 9a), resulting in the eventual increase in PFOA concentration at the shore before plateaus in concentrations for the simulations with higher sorption were reached (Figure 9b). Figure 9a demonstrates that the simulated PFOA concentrations at the downgradient PRB boundary just prior to breakthrough were 0.23 and 0.80 μg/L for the *K*_*d*_ = 1.2 and *K*_*d*_ = 2.4 mL/g scenarios, respectively. The higher PFOA concentrations shown at the shore for these two scenarios (Figure 9b) indicate that the installation of the CAC PRB would have resulted in a smaller mass flux reduction at the shore relative to the low *K*_*d*_ case. The degree of near‐term mass flux reduction that can be expected at coastal sites is sensitive to *K*_*d*_. For this reason, *K*_*d*_ is an important parameter that should be characterized at the remedial investigation stage if a CAC remedy is being considered for a coastal site with AFFF impacts.

In contrast to its effect on mass flux reduction, Figure 9a shows that PFOA breakthrough occurred at the downgradient boundary of the CAC zone at around the same time for all three cases. This indicates that *K*_*d*_ has relatively little influence on CAC longevity as breakthrough of PFOA in the CAC PRB is primarily controlled by the CAC loading (*f*_*cac*_) and CAC adsorption isotherm for PFOA.

### Case 3—Influence of Distance from CAC Zone to the Shore

When moving the CAC zone 5 m closer to the shore (*d*_inland_ = 10 m for the downgradient CAC boundary), the maximum (outward) groundwater velocity at the downgradient CAC boundary increased to 0.42 m/d, and the minimum (inward) velocity was −0.23 m/d. These velocities are about 20% higher in the outward and inward directions relative to the base case CAC location (*d*_inland_ = 15 m). In addition to higher velocities, moving the CAC zone location closer to the shore resulted in a slightly longer period when inward flow was occurring in each tidal cycle.

**Figure 10:**
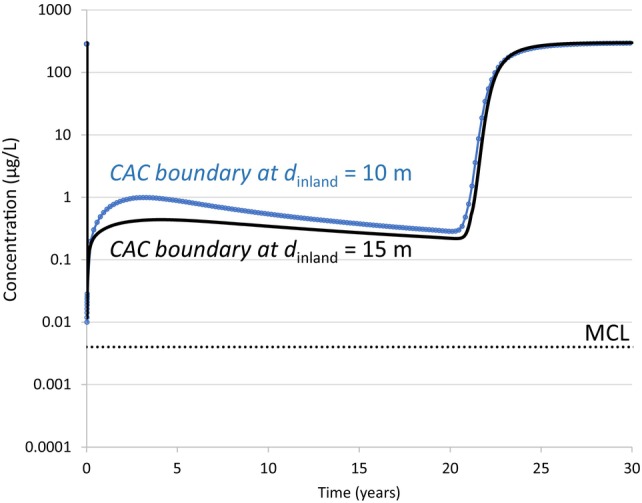
Simulated PFOA concentration versus time at the CAC downgradient boundary for the Base Case (*d*_inland_ = 15 m) and Case 3 (*d*_inland_ = 10 m).

The simulated PFOA concentration versus time at the downgradient CAC boundary for these two cases is shown in Figure 10. When the CAC zone was installed at a distance of 5 m closer to the shore, this resulted in a small increase in the PFOA accumulation at the downgradient CAC boundary (maximum of 1.0 μg/L compared to 0.44 μg/L for the base case). This indicates that moving the CAC zone 5 m closer than the base case does not have a substantial effect because the increase in the inward groundwater velocity was relatively small for this site scenario. In practice however, there is a higher risk of CAC particles being transported directly to the shore if the CAC zone is too close, so this should be considered when designing the placement of a CAC PRB.

### Case 4—Influence of K_f_

**Figure 11:**
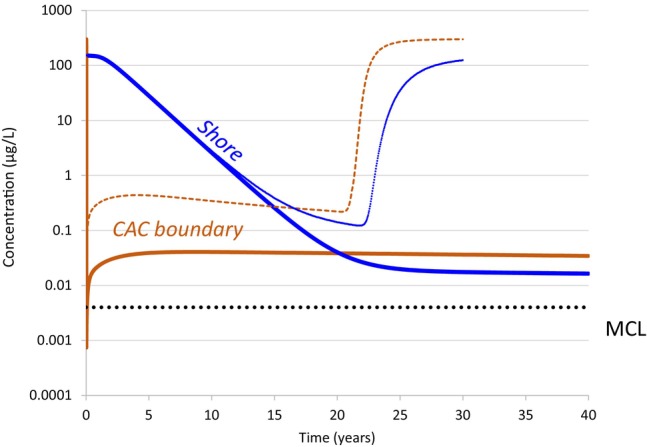
Simulated PFOA concentration versus time at the CAC downgradient boundary (orange) and at the shore (blue) for Case 4 [*K*_*f*_ = 1740 mg/kg (mg/L)^−*a*^] shown with solid lines; and the base case [*K*_*f*_ = 870 mg/kg (mg/L)^−*a*^] shown as a dashed line for the CAC downgradient boundary and a dotted line for the shore. The base case simulation was 30 years in duration, and Case 4 was 40 years due to the greater longevity of the CAC remedy.

Increasing *K*_*f*_ by a factor of two [from 870 to 1740 mg/kg (mg/L)^−*a*^] to account for potential enhanced adsorption due to high calcium and magnesium in the near‐shore environment, resulted in a corresponding increase in the CAC longevity by a factor of 2 (Figure 11). This direct correlation is consistent with the longevity model sensitivity analysis reported by Carey et al. (2022). Immediately after CAC injection, the post‐injection PFOA concentration in the CAC zone initially decreased by more than five orders of magnitude from 300 to 0.0009 μg/L. Figure 11 shows that the downgradient CAC boundary concentration then increased by close to two orders of magnitude (to 0.05 μg/L) because of the periodic inward groundwater velocity during high tide.

The shoreline concentration versus time for the higher *K*_*f*_ case declined relatively quickly (half‐life of 0.5 years), and then it leveled off at a concentration of about 0.02 μg/L 25 years after CAC injection. This plateau in PFOA concentration versus time at the shore is caused by the slow desorption of PFOA from the downgradient boundary, which is sustaining PFOA concentrations above the MCL for a long time. The stable shoreline concentration still represents a mass flux reduction of 99.993%.

### Case 5—Rate of Concentration Decline at the Shore for PFOA Versus PFOS

PFOS has a higher affinity than PFOA for adsorption to CAC (Carey et al. 2022; Hakimabadi et al. 2023) and NOM (McGuire et al. 2014; Xiao et al. 2017; Carey et al. 2019). While CAC longevity is substantially higher for PFOS, the rate of flushing of the higher mass of adsorbed PFOS downgradient of the CAC zone will result in a slower rate of decline in concentrations at the compliance boundary (i.e., at the shore). The base case simulation for PFOA was replicated for PFOS to compare post‐injection trends at the CAC boundary and at the shore for these two POCs.

The base case PFOA simulation incorporated a *K*_*d*_ of 1.2 mL/g based on an equivalent *f*_*oc*_ of 1.0%. The corresponding *K*_*d*_ for PFOS is 9.2 mL/g, resulting in PFOA and PFOS dimensionless retardation coefficients of 11 and 75, respectively. Carey et al. (2022) reported Freundlich isotherm parameters for PFOS based on groundwater sample collected at an AFFF‐impacted site as follows: *K*_*f*_ = 12,000 mg/kg (mg/L)^−*a*^, and *a* = 0.33. Hakimabadi et al. (2023) demonstrated that PFOS adsorption to CAC is enhanced with increasing ionic strength, similar to PFOA. Hakimabadi et al. (2023) were not able to estimate a Freundlich isotherm for PFOS at an ionic strength of 100 mM due to a lack of detected PFOS in solution. For this study, it is assumed that the increased PFOS *K*_*f*_ at an average near‐shore ionic strength of 84 mM has the same relative increase of 50% as was determined for PFOA. The hypothetical PFOS source concentration was specified to be the same as PFOA (i.e., 300 μg/L).

**Figure 12:**
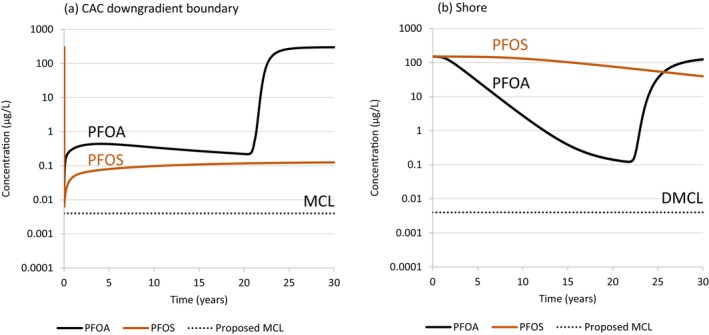
Simulated PFOA and PFOS concentration versus time at: (a) the CAC downgradient boundary; and (b) the shore for Case 1 (PFOA *K*_*d*_ = 1.2 mL/g and PFOS *K*_*d*_ = 9.2 mL/g).

A comparison of PFOA and PFOS concentration versus time trends at the CAC boundary and at the shore, for the base case scenario, is illustrated in Figure 12. (In this scenario, PFOA is simulated to breakthrough the CAC zone after 20 years, whereas PFOS breakthrough does not occur during the 40‐year simulation.) At the CAC boundary, the PFOS concentration 30 years after injection is 30 times higher than the MCL and appears to be continuing to increase at a slow rate. This represents the influence of tidally induced groundwater flow direction reversals at this downgradient CAC boundary. PFOA has a higher downgradient CAC boundary concentration but this is declining more quickly due to the faster rate of PFOA mass flushing and lower *K*_*d*_ in the region directly downgradient of the CAC zone. The PFOS simulation indicates that the influence of tidal fluctuations will result in a limit to the mass flux reduction that is attainable for PFOS in the longer term at the shore due to PFOS mass accumulation at the downgradient CAC boundary.

Figure 12 also shows that the rate of decline in PFOS concentrations at the shore is substantially slower than that for PFOA, which is expected because the PFOS *K*_*d*_ between the CAC zone and the shore that is about an order of magnitude higher than for PFOA. The concentration decline rates at the shore for PFOA and PFOS up to 20 years after CAC injection were calculated to be approximately 0.5 and 0.05 year^−1^, respectively; these correspond to decline half‐lives of 1.5 and 15 years, respectively. The PFOA mass flux reduction at the shore 20 years after CAC injection was 99.9%. It would take 10 times longer for PFOS to reach this same level of mass flux reduction. This demonstrates the importance of measuring PFAS *K*_*d*_ in the region between the CAC zone and a compliance boundary, to facilitate a prediction of the time it may take to reach a target mass flux reduction. Cai et al. (2022) and Navarro et al. (2022) determined that PFOS may have a significant fraction of irreversibly adsorbed mass in high *f*_*oc*_ aquifers. These model results assume that all PFOS mass is fully reversible; a significant irreversible sorbed fraction of PFOS will result in faster concentration declines at the shore than were simulated in this study. More research is needed to confirm the degree of irreversible PFOS adsorption expected to occur in the near‐shore environment at coastal sites.

## Conclusions and Recommendations

Hydrogeologic and geochemical settings were analyzed for a coastal site in the United States. A 3‐week tidal study was used to estimate the average tidal amplitude for seawater adjacent to the site (0.73 m). A simple application of an analytical solution for fluctuating heads in a tidal environment was used to calibrate the hydraulic conductivity for the near‐shore environment (2.6 m/d), by matching the modeled and observed tidal efficiency versus distance trends. This hydraulic conductivity is consistent with the fine‐grained sand with silt that comprises the artificial fill unit which is of interest for this study. The regional average hydraulic gradient in this unit was estimated to be 0.0075 m/m based on tidal monitoring.

The average near‐shore ionic strength is 84 mM, which was estimated to increase the adsorption of PFOA to CAC by about 50% relative to a non‐coastal site. The average concentrations of calcium and magnesium in the near‐shore environment were each 190 mg/L; these are expected to also increase the adsorption of PFOA to CAC but there is uncertainty in the magnitude of this effect, so this was conservatively not included in the base case scenario.

A one‐dimensional groundwater flow model was constructed and verified to represent the tidally influenced groundwater fluctuations in the artificial fill unit at the site. Flow model results show that tidally influenced groundwater fluctuations at the site occur up to *d*_inland_ of 125 m; however, the groundwater flow reversals (i.e., where groundwater velocity switches from outward to inward during the tidal cycle) only occurs up to *d*_inland_ of 40 m. This indicates that at this specific coastal site, a CAC zone installed at *d*_inland_ of more than 40 m will not be subject to reversals in the direction of groundwater flow.

A hypothetical scenario was constructed with a one‐dimensional reactive transport model (ISR‐MT3DMS) to evaluate the general effects of coastal hydrogeology and geochemistry on CAC in situ remediation of PFOA in the near‐shore environment. This modeling confirmed the hypothesis that tidally induced groundwater flow reversals near the shore result in the accumulation of PFOA at the downgradient CAC boundary. Slow desorption of PFOA from this downgradient CAC boundary may sustain downgradient plume concentrations above the cleanup criterion for a long time; however, after several decades there was consistently a large mass flux reduction (greater than 99.9%) downgradient of the CAC zone for all PFOA scenarios that were conducted in this modeling study. The degree of mass flux reduction that can be attained at the shore is strongly dependent on the PFOA *K*_*d*_ in the aquifer between the CAC zone and the shore. The longevity of a 6 m long CAC PRB downgradient of a high‐concentration PFOA source (300 μg/L) was predicted to be on the order of 20 to 40 years.

The rate of PFOS concentration decline at the shore was 10 times slower than for PFOA due to the higher mass of PFOS adsorbed to NOM downgradient from the CAC zone. This demonstrates the importance of characterizing apparent *K*_*d*_ for PFAS downgradient from a region where a PRB or pump‐and‐treat system is to be installed, so that realistic mass flux reduction targets can be estimated. The rate of PFAS decline at a coastal site compliance boundary will be substantially slower than at non‐coastal sites due to enhanced PFAS adsorption to NOM.

The findings from this study indicate that PFAS *K*_*d*_ should be characterized during the site investigation phase given the enhanced PFAS adsorption that occurs in near‐shore environments at coastal sites. A representative *K*_*d*_ may be estimated for a specific region at a site based on averaged results for multiple pairs of co‐located soil and groundwater samples collected under similar geochemical conditions. Fraction of organic carbon (*f*_*oc*_) should also be characterized in portions of the site which are not impacted by high organic chemical concentrations in soil, to facilitate an assessment of the extent to which geochemistry at coastal sites may be influencing *K*_*d*_ at varying distances inland. It is also recommended that additional isotherms be developed for PFAS‐CAC adsorption based on groundwater samples collected from near‐shore environments. At present, this is a major data gap with respect to predicting CAC longevity at coastal sites. The degree of reversibility of sorbed PFAS of concern to NOM is another data gap which requires further research for coastal sites.

Notably, the model simulations presented here suggest that short‐term remedial action objectives for coastal sites should be based on mass flux reduction targets rather than strict cleanup criteria which may be difficult to meet downgradient of a CAC zone in the near‐term.

Another data gap that warrants further study is the extent to which PFAS adsorption to CAC in the PRB exhibits rate‐limited behavior, particularly during the peaks and valleys of the tidally influenced groundwater velocity in the PRB.

## Supporting information


**Figure S1.** Modeled heads versus time based on average tidal cycle properties and the Ferris (1952) analytical solution.
**Figure S2.** Graphs of analytical results versus distance inland for monitoring wells at the coastal site.
**Figure S3.** Relationship between PFOS Kd and ionic strength in seawater. Kd data at various seawater dilutions were presented in Chen et al. (2012). The ionic strength was calculated as part of this data based on the assumption that seawater has an ionic strength of 700 mM, and freshwater has an ionic strength equal to the lowest I (7 mM) measured in groundwater at monitoring well MW‐9 at the coastal site.
**Figure S4.** Modeled heads at the right boundary representing simulated heads at a distance inland of 0.125 m. The black line with square symbols represents the MODFLOW simulated heads at the boundary grid cell with results at the start of each stress period; and the orange line represents the analytical solution results.
**Figure S5.** Modeled heads at distances inland of 15, 30, and 60 meters. The line series represent simulated results with the analytical solution, and the symbols represent results based on MODFLOW simulations.
**Figure S6.** PFOA concentration adsorbed to CAC versus ionic strength. The adsorbed concentrations were calculated based on an equilibrium aqueous concentration of 0.3 ug/L which is consistent with the batch test concentrations in Hakimabadi et al. (2023) batch tests. The Freundlich isotherms used to estimate the adsorbed concentration at each ionic strength are presented in Hakimabadi et al. (2023).
**Figure S7.** Simulated PFOA concentration contours at simulation times of 0, 2, 5, 10, 15, 20, 25, and 30 years after CAC injection. Note that the PFOA concentration does not decline below the EPA proposed MCL based on the simulated adsorption isotherm and fraction of colloidal activated carbon (fcac).
**Table S1.** Ranges of compressibility from Freeze and Cherry (1979) and Domenico and Mifflin (1965). The values associated with sand from Freeze and Cherry, and with loose sand from Domenico and Mifflin, were determined to be representative of the artificial fill hydrostratigraphic unit at the coastal site.
**Table S2.** Geochemistry analytical results for monitoring wells at the coastal site.
**Table S3.** Groundwater flow and reactive transport model input parameters.

## Data Availability

Research data are not shared.

## References

[gwat13456-bib-0001] Adamson, D.T. , P.R. Kulkarni , A. Nickerson , C.P. Higgins , J. Field , T. Schwichtenberg , C. Newell , and J.J. Kornuc . 2022. Characterization of relevant site‐specific PFAS fate and transport processes at multiple AFFF sites. Environmental Advances 7: 100167. 10.1016/j.envadv.2022.100167

[gwat13456-bib-0002] Adamson, D.T. , A. Nickerson , P.R. Kulkarni , C.P. Higgins , J. Popovic , J. Field , A. Rodowa , C. Newell , P. DeBlanc , and J.J. Kornuc . 2020. Mass‐based, field‐scale demonstration of PFAS retention within AFFF‐associated source areas. Environmental Science and Technology 54, no. 24: 15768–15777. 10.1021/acs.est.0c04472 33270425

[gwat13456-bib-0003] Cai, W. , D.A. Navarro , J. Du , G. Ying , B. Yang , M.J. McLaughlin , and R.S. Kookana . 2022. Increasing ionic strength and valency of cations enhance sorption through hydrophobic interactions of PFAS with soil surfaces. Science of the Total Environment 817: 152975. 10.1016/j.scitotenv.2022.152975 35026264

[gwat13456-bib-0004] Carey, G.R. , R.H. Anderson , P. Van Geel , R. McGregor , K. Soderberg , A. Danko , S.G. Hakimabadi , A.L.T. Pham , and M. Rebeiro‐Tunstall . 2023. Analysis of colloidal activated carbon alternatives for in situ remediation of a large PFAS plume and source area. Remediation Journal 34, no. 1: 1–18. 10.1002/rem.21772

[gwat13456-bib-0005] Carey, G.R. , S.G. Hakimabadi , M. Singh , R. McGregor , C. Woodfield , P.J. Van Geel , and A.L.‐T. Pham . 2022. Longevity of colloidal activated carbon for in situ PFAS remediation at AFFF‐impacted airport sites. Remediation Journal 33, no. 1: 3–23.

[gwat13456-bib-0006] Carey, G.R. , R. McGregor , A.L.‐T. Pham , B. Sleep , and S.G. Hakimabadi . 2019. Evaluating the longevity of a PFAS in situ colloidal activated carbon remedy. Remediation Journal 25: 17–31. 10.1002/rem.21593

[gwat13456-bib-0007] Carr, P.A. , and G.S. Van Der Kamp . 1969. Determining aquifer characteristics by the tidal method. Water Resources Research 5, no. 5: 1023–1031. 10.1029/WR005i005p01023

[gwat13456-bib-0008] Chen, H. , M. Reinhard , V.T. Nguyen , and K.Y.H. Gin . 2016. Reversible and irreversible sorption of perfluorinated compounds (PFCs) by sediments of an urban reservoir. Chemosphere 144: 1747–1753. 10.1016/j.chemosphere.2015.10.055 26521093

[gwat13456-bib-0009] Chen, H. , C. Zhang , Y. Yu , and J. Han . 2012. Sorption of perfluorooctane sulfonate (PFOS) on marine sediments. Marine Pollution Bulletin 64, no. 5: 902–906. 10.1016/j.marpolbul.2012.03.012 22472786

[gwat13456-bib-0010] Domenico, P.A. , and M.D. Mifflin . 1965. Water from low‐permeability sediments and land subsidence. Water Resources Research 1, no. 4: 563–576. 10.1029/WR001i004p00563

[gwat13456-bib-0011] Ferris, J.G. (1952) Cyclic Fluctuations of Water Level as a Basis for Determining Aquifer Transmissibility. *United States Geological Survey, Groundwater Notes – Hydraulics Section, Contribution* 1, April 1952. Washington, D.C.

[gwat13456-bib-0012] Freeze, R.A. , and J.A. Cherry . 1979. Groundwater, 624. Englewood Cliffs, NJ: Prentice‐Hall Inc.

[gwat13456-bib-0013] Hakimabadi, S.G. , A. Taylor , and A.L.‐T. Pham . 2023. Factors affecting the adsorption of per‐ and polyfluoroalkyl substances (PFAS) by colloidal activated carbon. Water Research 242: 120212. 10.1016/j.watres.2023.120212 37336180

[gwat13456-bib-0014] Harbaugh, A.W. , and M.G. McDonald . 1996. User's Documentation for MODFLOW‐96, an Update to the U.S. Geological Survey Modular Finite‐Difference Ground‐Water Flow Model. United States Geological Survey Open‐File Report 96‐485. Reston, Virginia.

[gwat13456-bib-0015] Irvine, D.J. , A.D. Werner , Y. Ye , and A. Jazayeri . 2021. Upstream dispersion in solute transport models: A simple evaluation and reduction methodology. Groundwater 59, no. 2: 287–291. 10.1111/gwat.13036 32754918

[gwat13456-bib-0033] Jacob, C.E. 1950. Flow of groundwater in Engineering Hydraulics, ed. H. Rouse , 321–386. New York: John Wiley.

[gwat13456-bib-0016] Leeson, A. , T. Thompson , H.F. Stroo , R.H. Anderson , J. Speicher , M.A. Mills , J. Willey , C. Coyle , R. Ghosh , C. Lebron , and C. Patton . 2021. Identifying and managing aqueous film‐forming foam‐derived per‐and polyfluoroalkyl substances in the environment. Environmental Toxicology and Chemistry 40, no. 1: 24–36. 10.1002/etc.4894 33026660 PMC7839684

[gwat13456-bib-0017] McGregor, R. 2023. The in situ treatment of PFAS within porewater at the air‐water interface of a PFAS source zone. Remediation Journal 33: 265–278.

[gwat13456-bib-0018] McGregor, R. 2020a. Six pilot‐scale studies evaluating the *in situ* treatment of PFAS in groundwater. Remediation Journal 30, no. 3: 39–50. 10.1002/rem.21653

[gwat13456-bib-0019] McGregor, R. 2020b. Distribution of colloidal and powdered activated carbon for the *in situ* treatment of groundwater. Journal of Water Resource and Protection 12: 1001–1018.

[gwat13456-bib-0020] McGregor, R. 2018. In situ treatment of PFAS‐impacted groundwater using colloidal activated carbon. Remediation Journal 28: 33–41. 10.1002/rem.21558

[gwat13456-bib-0021] McGregor, R. , and L. Benevenuto . 2021. The effect of heterogeneity on the distribution and treatment of PFAS in a complex geologic environment. Frontiers in Environmental Chemistry 2: 729779. 10.3389/fenvc.2021.729779

[gwat13456-bib-0022] McGregor, R. , and Y. Zhao . 2021. The in situ treatment of TCE and PFAS in groundwater within a silty sand aquifer. Remediation Journal 31, no. 2: 1–11. 10.1002/rem.21675

[gwat13456-bib-0023] McGuire, M.E. , C. Schaefer , T. Richards , W.J. Backe , J.A. Field , E. Houtz , D.L. Sedlak , J.L. Guelfo , A. Wunsch , and C.P. Higgins . 2014. Evidence of remediation‐induced alteration of subsurface poly‐ and perfluoroalkyl substance distribution at a former firefighter training Ara. Environmental Science and Technology 48, no. 12: 6644–6652. 10.1021/es5006187 24866261

[gwat13456-bib-0024] Mole, R.A. , A.C. Velosa , G.R. Carey , X. Liu , G. Li , D. Fan , A. Danko , and G. Lowry . 2023. Groundwater solutes influence the adsorption of short‐chain perfluoroalkyl acids (PFAA) to colloidal activated carbon and impact performance for in situ groundwater remediation. Water Research 474, no. 5: 1–10. 10.1016/j.jhazmat.2024.134746 38850952

[gwat13456-bib-0025] Navarro, D.A. , D.P. Oliver , S.L. Simpson , and R.S. Kookana . 2022. Organic carbon and salinity affect desorption of PFAS from estuarine sediments. Journal of Soils and Sediments 22, no. 4: 1302–1314.

[gwat13456-bib-0026] Oliver, D.P. , D.A. Navarro , J. Baldock , S.L. Simpson , and R.S. Kookana . 2020. Sorption behaviour of per‐ and polyfluoroalkyl substances (PFASs) as affected by the properties of coastal estuarine sediments. Science of the Total Environment 720: 137263. 10.1016/j.scitotenv.2020.137263 32145609

[gwat13456-bib-0027] Porewater Solutions . 2022. ISR‐MT3DMS Users Guide for Simulating Multispecies Reactive Transport. Ottawa, ON.

[gwat13456-bib-0028] Serfes, M.E. 1991. Determining the mean hydraulic gradient of ground water affected by tidal fluctuations. Ground Water 29, no. 4: 549–555. 10.1111/j.1745-6584.1991.tb00546.x

[gwat13456-bib-0029] United States Environmental Protection Agency (USEPA) . 1998. Technical protocol for evaluating natural attenuation of chlorinated solvents in ground water. USEPA, Report EPA/600/R‐98/128. Washington, D.C.

[gwat13456-bib-0030] Xiao, X. , B.A. Ulrich , B. Chen , and C.P. Higgins . 2017. Sorption of poly‐ and perfluoroalkyl substances (PFASs) relevant to aqueous film‐forming foam (AFFF)‐impacted groundwater by biochars and activated carbon. Environmental Science and Technology 51, no. 11: 6342–6351. 10.1021/acs.est.7b00970 28582977

[gwat13456-bib-0031] Yin, C. , C.G. Pan , S.K. Xiao , Q. Wu , H.M. Tan , and K. Yu . 2022. Insights into the effects of salinity on the sorption and desorption of legacy and emerging per‐ and polyfluoroalkyl substances (PFASs) on marine sediments. Environmental Pollution 300: 118957. 10.1016/j.envpol.2022.118957 35124123

[gwat13456-bib-0032] You, C. , C. Jia , and G. Pan . 2010. Effect of salinity and sediment characteristics on the sorption and desorption of perfluorooctane sulfonate at sediment‐water interface. Environmental Pollution 158, no. 5: 1343–1347. 10.1016/j.envpol.2010.01.009 20181418

