# Supplementary material for: Modeling the Influence of Coastal Site Characteristics on PFAS in Situ Remediation
Source: Ground Water. 2024 Dec 11;63(2):175–91. doi: 10.1111/gwat.13456 (PMC11875043; doi:10.1111/gwat.13456)
Supplement: Supplementary file 1 — Figure S1. Modeled heads versus time based on average tidal cycle properties and the Ferris (1952) analytical solution. Figure S2. Graphs of analytical results versus distance inland for monitoring wells at the coastal site. Figure S3. Relationship between PFOS Kd and ionic strength in seawater. Kd data at various seawater dilutions were presented in Chen et al. (2012). The ionic strength was calculated as part of this data based on the assumption that seawater has an ionic strength of 700 mM, and freshwater has an ionic strength equal to the lowest I (7 mM) measured in groundwater at monitoring well MW‐9 at the coastal site. Figure S4. Modeled heads at the right boundary representing simulated heads at a distance inland of 0.125 m. The black line with square symbols represents the MODFLOW simulated heads at the boundary grid cell with results at the start of each stress period; and the orange line represents the analytical solution results. Figure S5. Modeled heads at distances inland of 15, 30, and 60 meters. The line series represent simulated results with the analytical solution, and the symbols represent results based on MODFLOW simulations. Figure S6. PFOA concentration adsorbed to CAC versus ionic strength. The adsorbed concentrations were calculated based on an equilibrium aqueous concentration of 0.3 ug/L which is consistent with the batch test concentrations in Hakimabadi et al. (2023) batch tests. The Freundlich isotherms used to estimate the adsorbed concentration at each ionic strength are presented in Hakimabadi et al. (2023). Figure S7. Simulated PFOA concentration contours at simulation times of 0, 2, 5, 10, 15, 20, 25, and 30 years after CAC injection. Note that the PFOA concentration does not decline below the EPA proposed MCL based on the simulated adsorption isotherm and fraction of colloidal activated carbon (fcac). Table S1. Ranges of compressibility from Freeze and Cherry (1979) and Domenico and Mifflin (1965). The values associated with sand [file GWAT-63-175-s001.pdf]

Supporting Information

Modeling the Influence of Coastal Site Characteristics on PFAS *In Situ* Remediation

Grant R. Carey
Corresponding author: Porewater Solutions, 2958 Barlow Crescent, Ottawa, Ontario K0A 1T0; 613-832-1737; gcarey@porewater.com

Anthony Danko
Naval Facilities Engineering and Expeditionary Warfare Center, San Diego, CA; Danko, anthony.s.danko.civ@us.navy.mil

Anh Le-Tuan Pham
University of Waterloo, Waterloo, Ontario; anh.pham@uwaterloo.ca

Keir Soderberg
S.S. Papadopulos & Associates, Rockville, MD; keirs@sspa.com

Beth Hoagland
S.S. Papadopulos & Associates, Rockville, MD; bhoagland@sspa.com

Brent Sleep
University of Toronto, Toronto, Ontario; brent.sleep@utoronto.ca

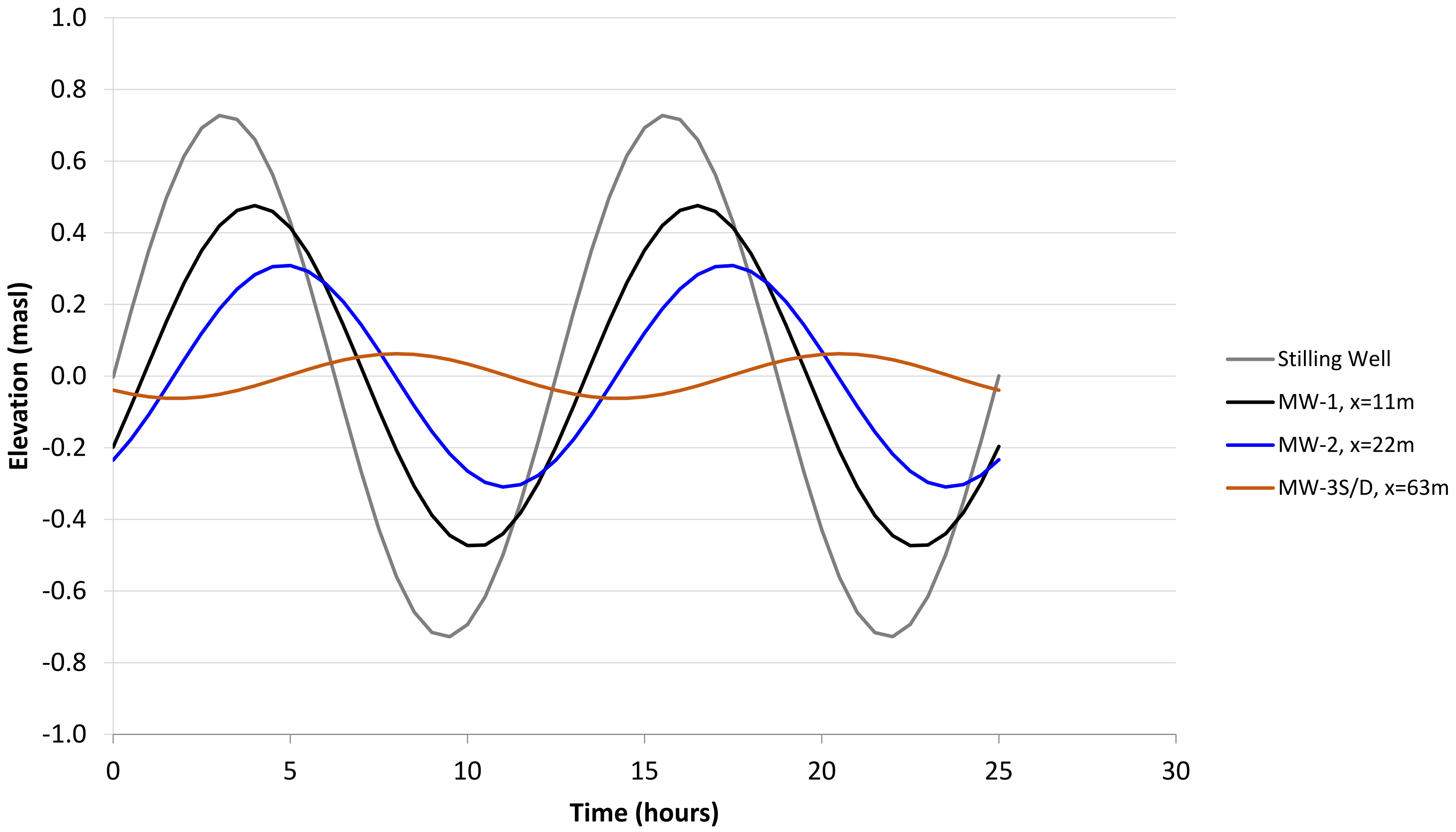

Figure SI-1. Modeled heads versus time based on average tidal cycle properties and the Ferris (1952) analytical solution.

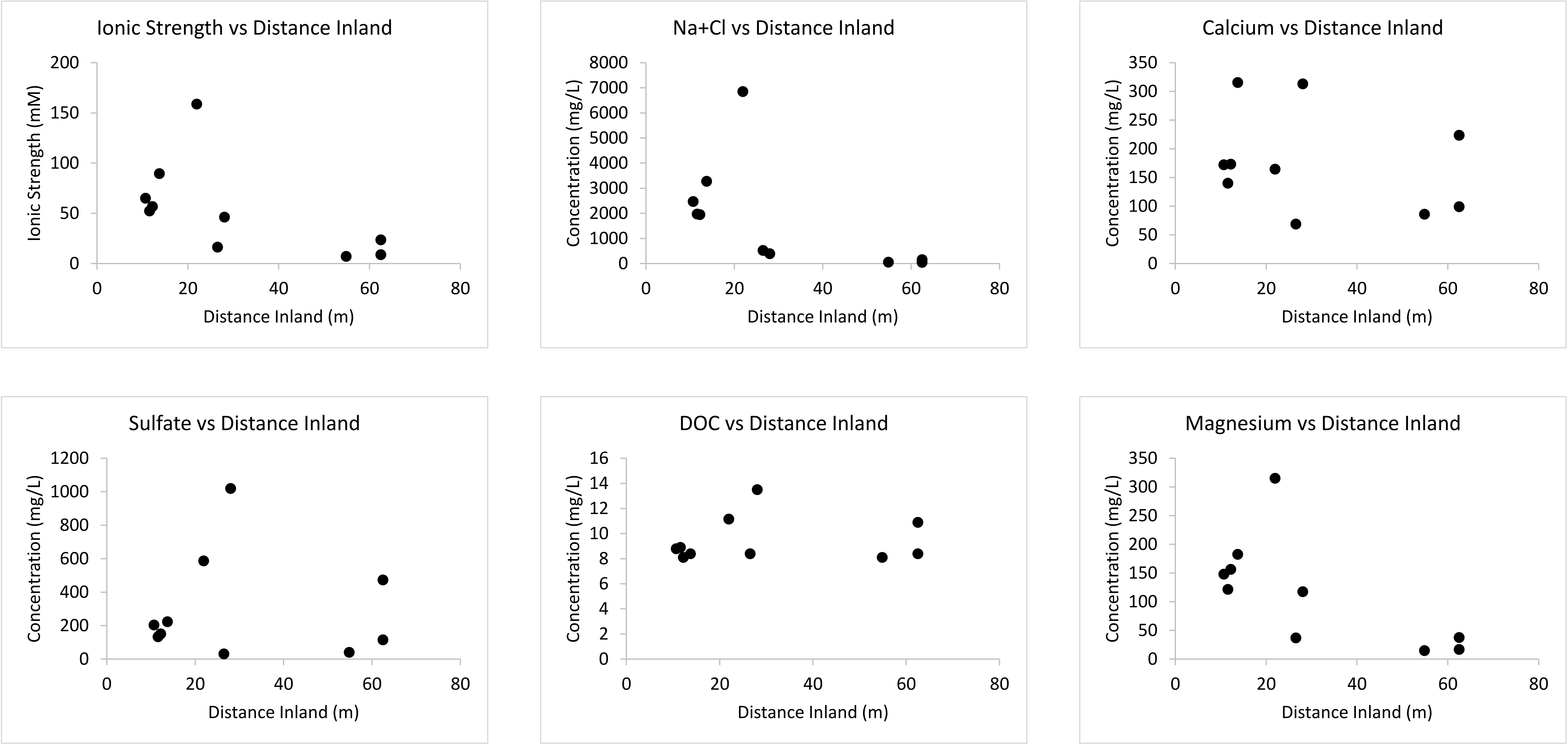

Figure SI-2. Graphs of analytical results versus distance inland for monitoring wells at the coastal site.

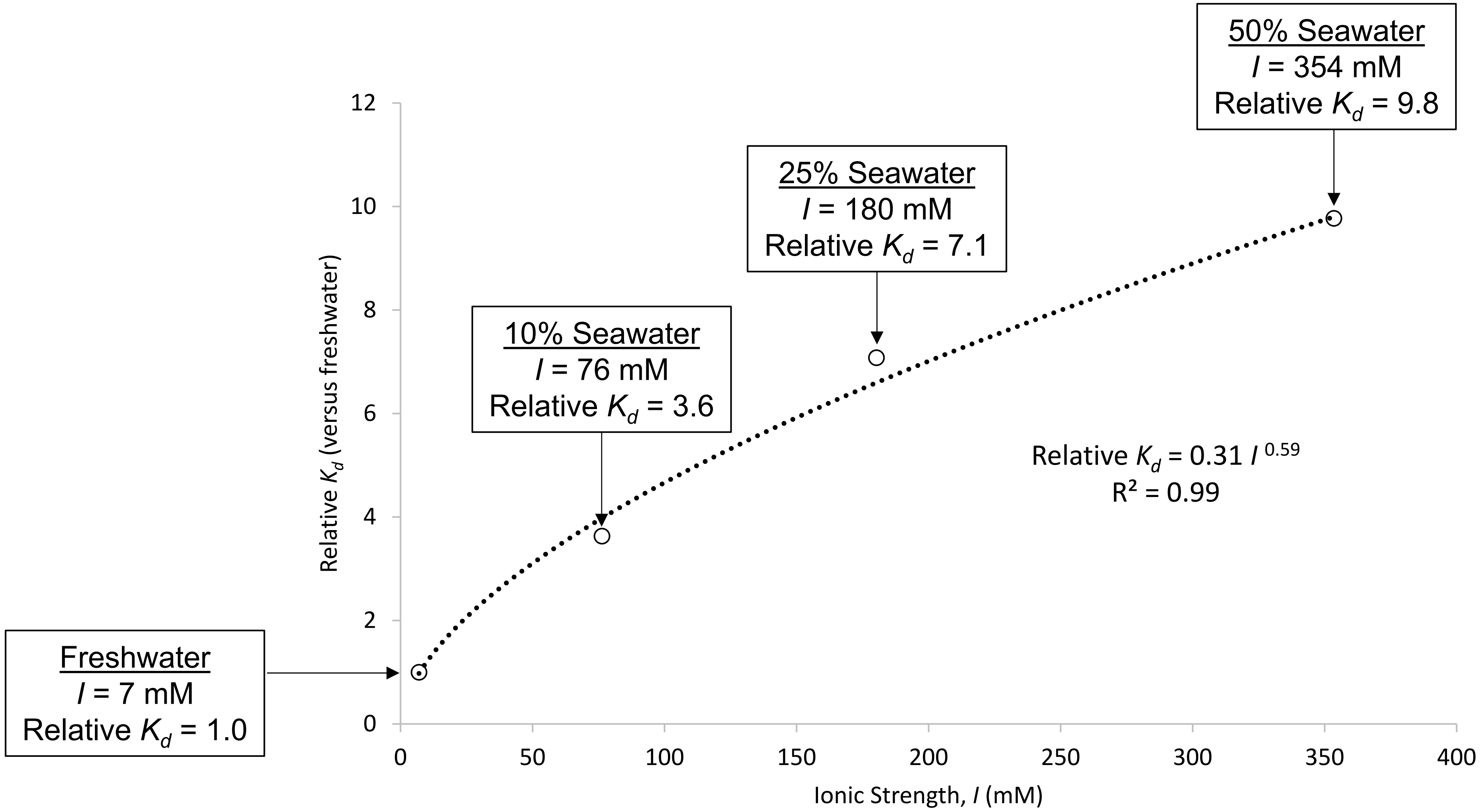

Figure SI-3. Relationship between PFOS K_d and ionic strength in seawater. *Kd* data at various seawater dilutions were presented in Chen et al. (2012). The ionic strength was calculated as part of this data based on the assumption that seawater has an ionic strength of 700 mM, and freshwater has an ionic strength equal to the lowest I (7 mM) measured in groundwater at monitoring well MW-9 at the coastal site.

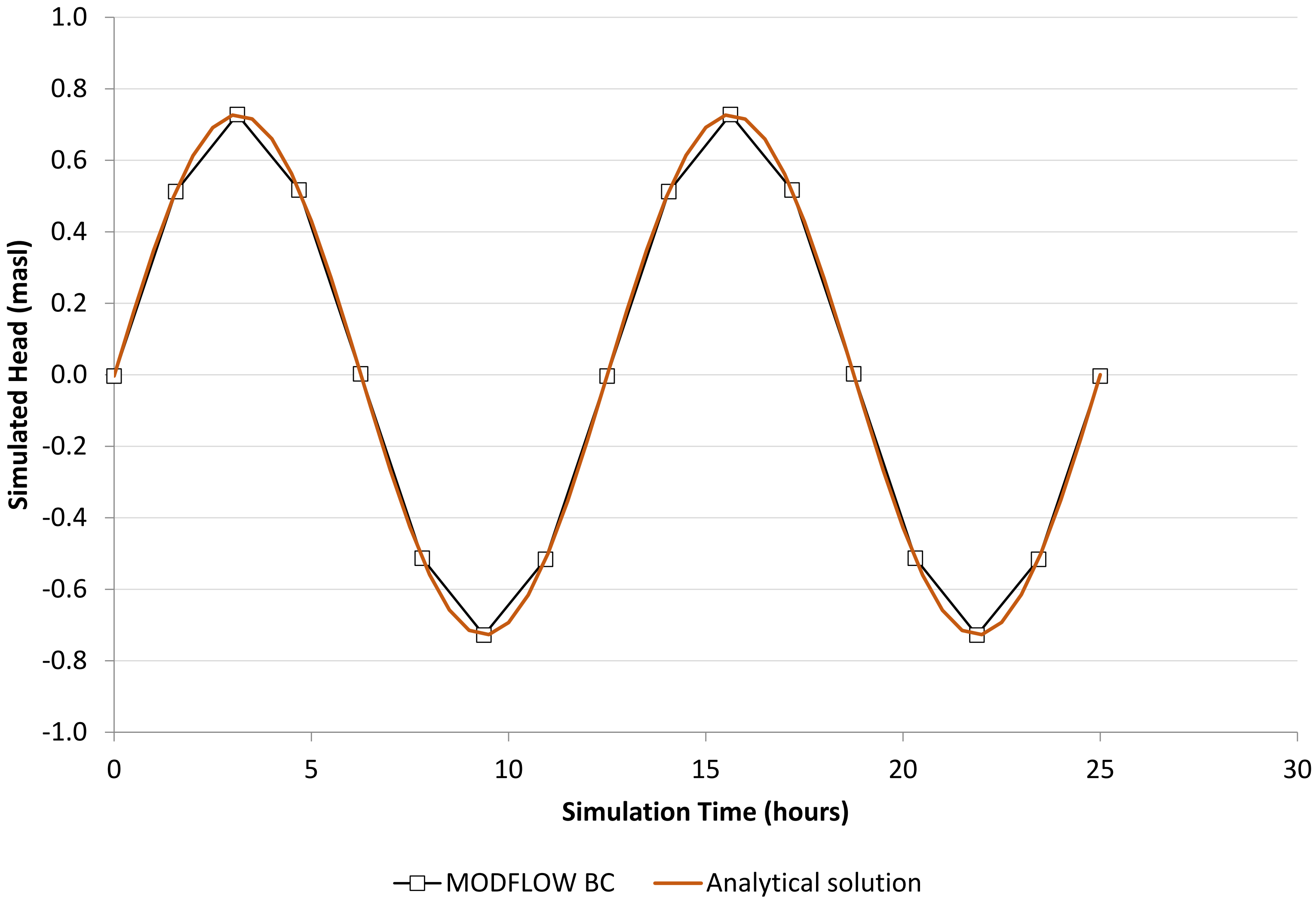

Figure SI-4. Modeled heads at the right boundary representing simulated heads at a distance inland of 0.125 m. The black line with square symbols represents the MODFLOW simulated heads at the boundary grid cell with results at the start of each stress period; and the orange line represents the analytical solution results.

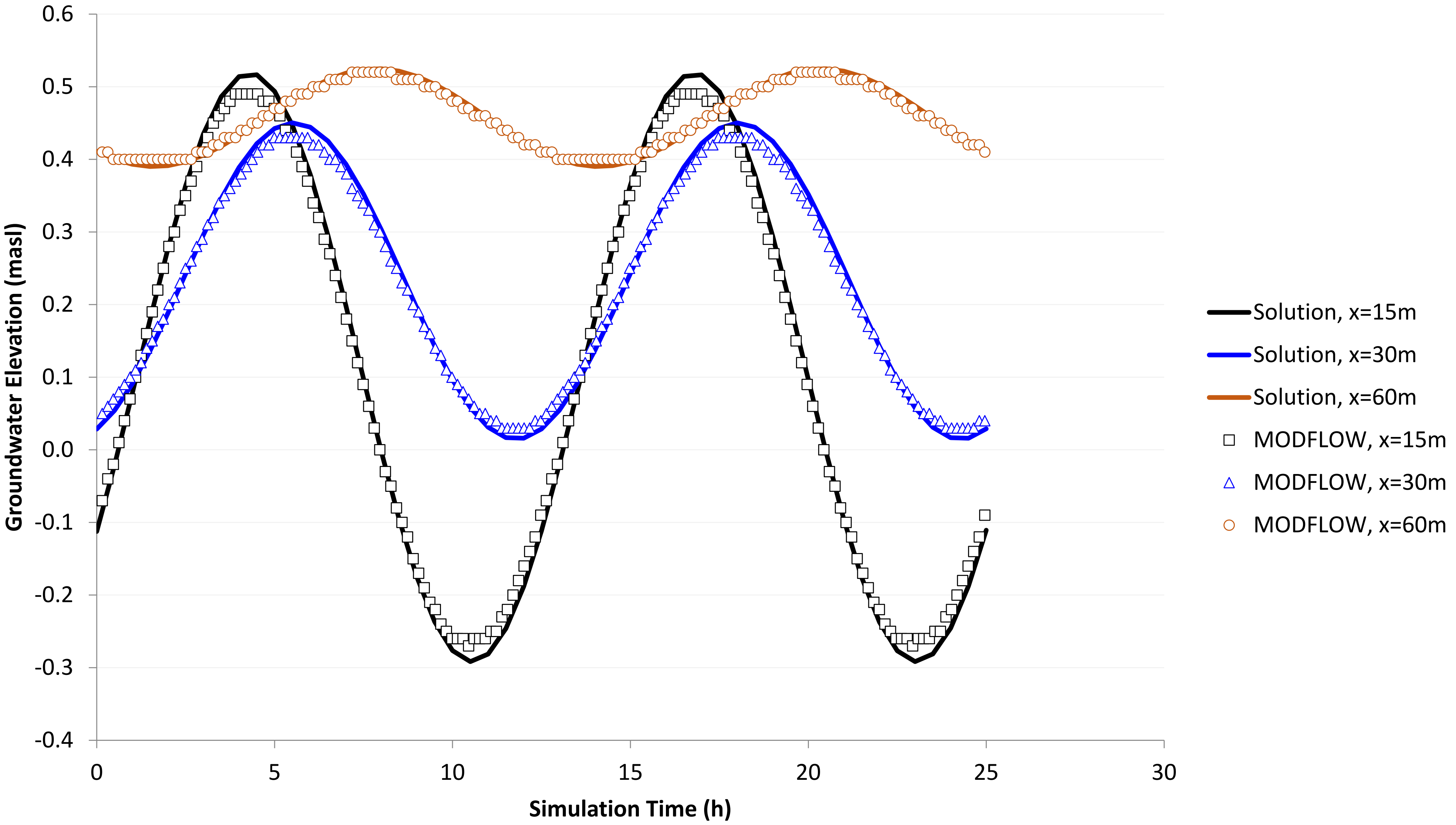

Figure SI-5. Modeled heads at distances inland of 15, 30, and 60 meters. The line series represent simulated results with the analytical solution, and the symbols represent results based on MODFLOW simulations.

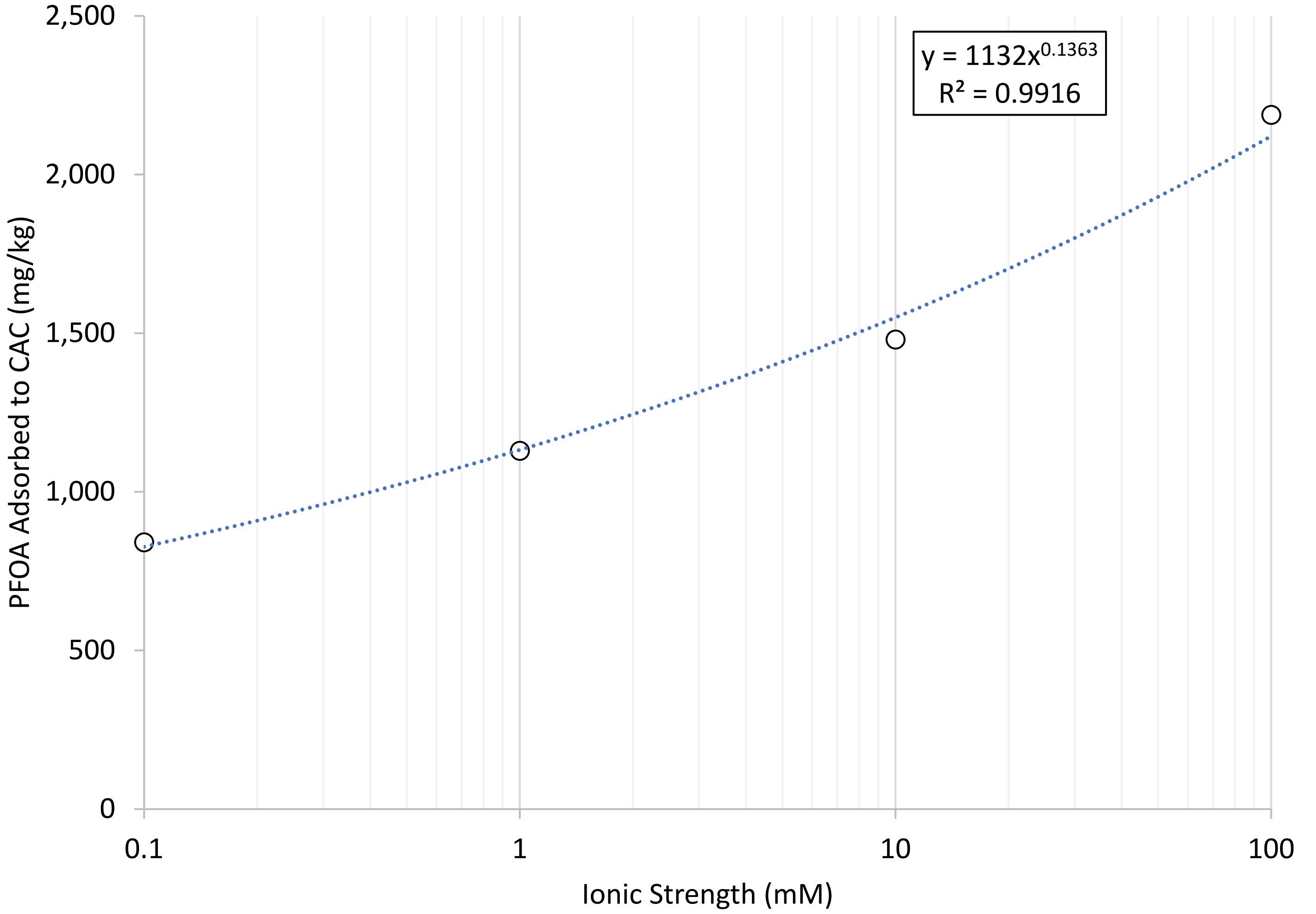

Figure SI-6. PFOA concentration adsorbed to CAC versus ionic strength. The adsorbed concentrations were calculated based on an equilibrium aqueous concentration of 0.3 ug/L which is consistent with the batch test concentrations in Hakimabadi et al. (2023) batch tests. The Freundlich isotherms used to estimate the adsorbed concentration at each ionic strength are presented in Hakimabadi et al. (2023).

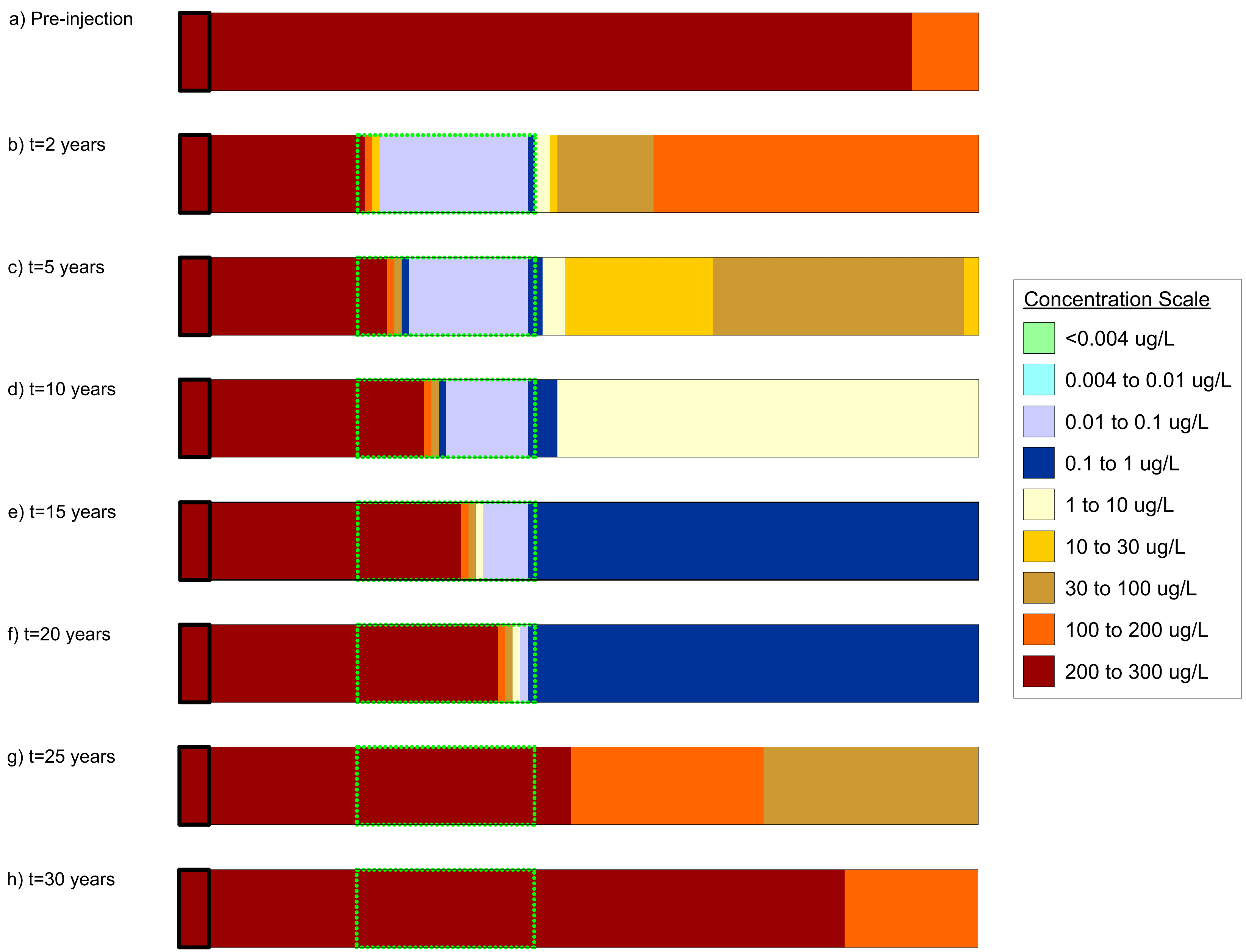

Figure SI-7. Simulated PFOA concentration contours at simulation times of 0, 2, 5, 10, 15, 20, 25, and 30 years after CAC injection. Note that the PFOA concentration does not decline below the EPA proposed MCL based on the simulated adsorption isotherm and fraction of colloidal activated carbon (f_{cac}).

Table SI-1. Ranges of compressibility from Freeze and Cherry (1979) and Domenico and Mifflin (1965). The values associated with sand from Freeze and Cherry, and with loose sand from Domenico and Mifflin, were determined to be representative of the artificial fill hydrostratigraphic unit at the coastal site.

a) Ranges of compressibility from Freeze and Cherry, 1979 - p. 54, Table 2.5

	Compressibility (Pa^{-1})		Specific Storage (ft^{-1})	
**Lithology**	**Low**	**High**	**Low**	**High**
clay	1.0E-08	1.0E-06	3.0E-05	3.0E-03
sand	1.0E-09	1.0E-07	3.5E-06	3.0E-04
gravel	1.0E-10	1.0E-08	8.3E-07	3.0E-05
fractured rock	1.0E-10	1.0E-08	8.3E-07	3.0E-05
sound rock	1.0E-11	1.0E-09	5.6E-07	3.5E-06
water	4.4E-10			

b) Ranges of compressibility from Domenico and Mifflin, 1965

	Compressibility (Pa^{-1})		Specific Storage (ft^{-1})	
**Lithology**	**Low**	**High**	**Low**	**High**
Plastic clay	2.0E-06	2.6E-07	6.0E-03	7.8E-04
Stiff clay	2.6E-07	1.3E-07	7.8E-04	3.9E-04
Medium-hard clay	1.3E-07	6.9E-08	3.9E-04	2.1E-04
Loose sand	1.0E-07	5.2E-08	3.0E-04	1.6E-04
Dense sand	2.0E-08	1.3E-08	6.0E-05	3.9E-05
Dense, sandy gravel	1.0E-08	5.2E-09	3.0E-05	1.6E-05
Rock, fissured	6.9E-10	3.3E-10	2.6E-06	1.5E-06
Rock, sound		3.3E-10	5.3E-07	1.5E-06

Table SI-2. Geochemistry analytical results for monitoring wells at the coastal site.

Location	Inland Distance (m)	Mid-Point Depth (m bgs)	Ionic Strength (mM)	Result (mg/L)										
				Alkalinity	Na+Cl	Chloride	Potassium	Sodium	Calcium	Magnesium	Sulfate	DOC	TOC	TSS
MW-1	11	3.5	65	349	2472	1670	54.4	802	172	148	204	8.8	8.9	9.2
MW-4	12	3.5	52	373	1968	1280	54.7	688	140	121.5	134	8.9	8.8	14.1
MW-5	12	3.5	57	448	1950.5	1300	62.35	650.5	173	156.5	151	8.1	8	3.1
MW-6	14	3.8	89	351	3275	2080	46.75	1195	315.5	182.5	223	8.4	8.5	8.7
MW-2	22	3.5	159	481	6847.5	4345	126	2502.5	164.5	315	587	11.15	11.2	4.05
MW-7	27	3.5	16	390	525	310	25.95	215	68.75	36.9	31.1	8.4	8.6	25.9
MW-8	28	3.5	46	515	388.5	126	48.05	262.5	313	117.5	1020	13.5	13.8	22.3
MW-9	55	3.5	7	279	57.35	23.8	7.795	33.55	85.9	14.7	40.3	8.1	7.9	3.8
MW-3S	63	2.7	9	232	46.15	13.9	14.1	32.25	99.15	16.8	115	8.4	8	4.8
MW-3D	63	4.0	24	387	150.8	35.8	28.5	115	223.5	37.75	473	10.9	10.7	7.1

Table SI-3. Groundwater flow and reactive transport model input parameters.

MODFLOW Simulation		
**Property**	**Units**	**Value**
Average tidal amplitude	m	0.73
Tidal period	hours	12.5
Hydraulic conductivity	m/day	2.6
Specific storage	m^{-1}	$6x10^{-4}$
Saturated thickness	m	3
Infiltration rate	m/day	0

ISR-MT3DMS Base Case Simulation (PFOA)		
**Property**	**Units**	**Value**
Effective porosity	m^3/m^3	0.2
Soil dry bulk density	g/mL	1.6
Longitudinal dispersivity	m	Inside CAC zone: 0.2;<br>Outside CAC zone: 2
Source concentration	mg/L	300
Kd	mL/g	1.2
Freundlich K_f	$(mg/kg)(mg/L)^{-a}$	870
Freundlich a	dimensionless	0.25
